# A Horizontally Transferred Plant Fatty Acid Desaturase Gene Steers Whitefly Reproduction

**DOI:** 10.1002/advs.202306653

**Published:** 2023-12-25

**Authors:** Cheng Gong, Zhaojiang Guo, Yuan Hu, Zezhong Yang, Jixing Xia, Xin Yang, Wen Xie, Shaoli Wang, Qingjun Wu, Wenfeng Ye, Xuguo Zhou, Ted C. J. Turlings, Youjun Zhang

**Affiliations:** ^1^ State Key Laboratory of Vegetable Biobreeding Department of Plant Protection Institute of Vegetables and Flowers Chinese Academy of Agricultural Sciences Beijing 100081 China; ^2^ Institute of Plant Protection Tianjin Academy of Agricultural Sciences Tianjin 300381 China; ^3^ Laboratory of Fundamental and Applied Research in Chemical Ecology Institute of Biology University of Neuchâtel Neuchâtel CH‐2000 Switzerland; ^4^ Department of Entomology University of Kentucky Lexington KY 40546‐0091 USA

**Keywords:** Δ12 desaturase, *Bemisia tabaci*, horizontal gene transfer, polyunsaturated fatty acids, reproduction

## Abstract

Polyunsaturated fatty acids (PUFAs) are essential nutrients for all living organisms. PUFA synthesis is mediated by Δ12 desaturases in plants and microorganisms, whereas animals usually obtain PUFAs through their diet. The whitefly *Bemisia tabaci* is an extremely polyphagous agricultural pest that feeds on phloem sap of many plants that do not always provide them with sufficient PUFAs. Here, a plant‐derived Δ12 desaturase gene family *BtFAD2* is characterized in *B. tabaci* and it shows that the *BtFAD2‐9* gene enables the pest to synthesize PUFAs, thereby significantly enhancing its fecundity. The role of *BtFAD2‐9* in reproduction is further confirmed by transferring the gene to *Drosophila melanogaster*, which also increases the fruit fly's reproduction. These findings reveal an extraordinary evolutionary scenario whereby a phytophagous insect acquired a family of plant genes that enables it to synthesize essential nutrients, thereby lessening its nutritional dependency and allowing it to feed and reproduce on many host plants.

## Graphical abstract

The study reveals that the whitefly *Bemisia tabaci* has acquired a family of plant genes, *BtFAD2*, that provide it with the unique ability to synthesize PUFAs by itself, enhancing its fecundity. The results imply a divergent evolutionary scenario by which this extremely phytophagous insect has acquired plant genes for key physiological functions and sheds light on the traits that allow for the insect's exceptional fecundity on its vast spectrum of host plants. Understanding the molecular mechanisms behind these traits can be crucial for managing this global pest.

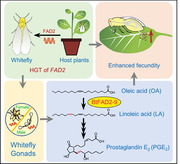

## 1 Introduction

Polyunsaturated fatty acids (PUFAs), which contain multiple double bonds between carbon atoms, are essential nutrients increasingly recognized as important to the survival of all organisms.^[^ ^1^ ^]^ PUFAs not only represent building blocks of biological membranes but also provide reservoirs of metabolic energy and serve as precursors for highly bioactive molecules such as prostaglandin E_2_ (PGE_2_).^[^ ^2^ ^]^ In mammals, PUFAs are associated with various physiological processes, including those in cardiovascular and central nervous systems,^[^ ^3^ ^]^ whereas in insects PUFAs are mainly involved in pheromone biosynthesis, cuticle formation, and immunity processes.^[^ ^4^ ^]^ PUFAs also contribute to the synthesis of reproductive tissues and can alter reproductive function and fertility.^[^ ^5^ ^]^ For example, PUFAs are involved in the development of mammalian testes and ovaries, whilst playing an important role in insect egg production and egg‐laying behavior.^[^ ^4^, ^6^ ^]^ Therefore, the acquisition of PUFAs by organisms is of key importance for their metabolic activities, especially those related to reproduction.

The complex biosynthesis of PUFAs requires the participation of multiple catalytic enzymes,^[^ ^7^ ^]^ among which Δ12 desaturase is the key enzyme that catalyzes the transformation of monounsaturated fatty acids (MUFAs) into PUFAs. However, the absence of enzymes like Δ12 desaturase in most organisms results in widely divergent PUFA acquisition capacities among species.^[^ ^8^ ^]^ The current dogma is that photosynthetic plants, heterotrophic protists, and bacteria account for most of the natural PUFA production because they have the enzymatic components necessary for de novo synthesis, unlike higher trophic organisms.^[^ ^9^ ^]^ Most animals acquire PUFAs through their diet in order to satisfy their requirements for essential fatty acids. However, exceptions have been reported for arthropods, including acarid mites (Acari), copepods (Crustacea), springtails (Collembola), the house cricket *Acheta domesticus* (Orthoptera), termites (Blattodea), the red flour beetle *Tribolium castaneum* (Coleoptera), the soldier beetle *Chauliognathus lugubris* (Coleoptera), a parasitic wasp *Nasonia vitripennis* (Hymenoptera), and the whitefly *Bemisia tabaci* (Hemiptera), all being able to biosynthesize PUFAs.^[^ ^10^ ^]^ Recent research has also revealed genes that encode key enzymes for PUFA synthesis in 80 invertebrate species, including several terrestrial arthropods such as *Locusta migratoria* (Orthoptera), *Sminthurus viridis* (Collembola) and *B. tabaci* (Hemiptera).^[^ ^11^ ^]^

The whitefly, *B. tabaci* (Gennadius), is a species complex of at least 30 cryptic species, some of which (e.g., Mediterranean [MED] and Middle East‐Asia Minor 1 [MEAM1]) are among the most devastating crop pests worldwide.^[^ ^12^ ^]^ Whiteflies damage plants by sucking plant phloem sap and transmitting plant viruses.^[^ ^13^ ^]^ *B. tabaci* is extremely polyphagous and is known to attack more than 600 plant species and shows exceptional host adaptability.^[^ ^14^ ^]^ Achieving a sufficient nutrient supply from such a broad range of host plants must confront the whitefly with an exceptional nutritional challenge.^[^ ^15^ ^]^ The way that the whitefly obtains essential nutrients has been studied to some extent,^[^ ^16^ ^]^ but their ability to synthesize PUFA's remains poorly understood.

Using a combination of chemical and molecular research tools, as well as insect performance assays, we characterized the horizontally transferred plant *BtFAD2* gene family, which encodes Δ12 desaturase‐like enzymes in *B. tabaci*. Of this gene family, the *BtFAD2‐9* gene is shown to be specifically expressed in the gonads and to play an important role in whitefly reproduction. Our discovery reveals a key molecular mechanism that makes *B. tabaci* far less dependent on the nutritional quality of their numerous host plants. These findings can be the basis for the development of new strategies to control this exceedingly important pest.

## 2 Results

### 2.1 Identification and Characterization of *BtFAD2* Gene Family in Whitefly

**Figure 1:**
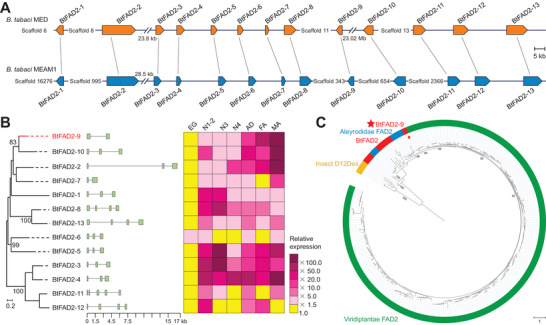
Genome‐wide characterization of the *BtFAD2* gene family in *B. tabaci*. A) Synteny analysis of 13 *FAD2* genes among *B. tabaci* MED and MEAM1. B) Genes are organized according to their phylogenetic analysis constructed by the Bayesian‐based phylogenetic analysis based on the optimized WAG + G model at 700 aligned amino acid positions. Gene architectures are shown by a green rectangle (exon) and a black line (intron). The constitutive transcription profiles of *BtFAD2* genes in eggs (EG), 1st‐ and 2nd‐instar nymphs (N1‐2), 3rd‐instar nymphs (N3), 4th‐instar nymphs (N4), adults (AD), female adults (FA) and male adults (MA) as determined by qPCR. For each gene, the expression fold changes are color‐coded according to the gradient, magenta rectangles indicate significant up‐regulation (ratio > 1.5‐fold), while yellow rectangles indicate no significant transcription variations. Data are presented as means, *n* = 3 biologically independent samples. C) Bayesian‐based phylogenetic analysis of BtFAD2 with JTT + I + G model at 859 aligned amino acid positions. After midpoint rooting, evolutionary branches were formed by 13 FAD2 proteins, within a group of Aleyrodinae FAD2 proteins. Only the Bayesian posterior probabilities (× 100) at phylogenetically important nodes are shown. BtFAD2‐9 is indicated by a red star.

To investigate the mechanism of nutrient synthesis in whiteflies, we constructed a schematic diagram of a general de novo PUFA biosynthetic pathway based on fatty acid classification and available data in KEGG (Kyoto Encyclopedia of Genes and Genomes) (Figure S1, Supporting Information). Based on a preliminary survey of *B. tabaci* MED genes related to constructed pathways and on two previous studies,^[^ ^11^, ^17^ ^]^ we identified the *BtFAD2* gene family in the *B. tabaci* MED genome. This gene family consists of 13 genes cloned from *B. tabaci* by specific PCR primers (Figure S2B and Table S1, Supporting Information). These genes are distributed across four scaffolds of the *B. tabaci* MED genome and share synteny between the MED and MEAM1 genomes, and some of them form gene clusters and have largely undergone tandem gene duplication during genome evolution (**Figure** **1**A). The observed synteny was also found in the *B. tabaci* MED chromosome and *B. tabaci* SSA genome (Figure S2A, Supporting Information).^[^ ^17^, ^18^ ^]^ Although *FAD2* genes in plants usually have only one exon, the *BtFAD2* genes exhibit 2–4 exons (Figure 1B). All BtFAD2 proteins carry three conserved histidine box motifs (HXCGH motif, HXXHH motif, and HXXHH motif), which are conserved domains of plant Δ12 desaturase proteins (Figure S2C,D, Supporting Information).^[^ ^19^ ^]^ BtFAD2 proteins share 20–70% sequence similarity, but uniformly exhibit > 30% similarity compared to the only *FAD2* gene in the model plant *Arabidopsis thaliana*, particularly 68% for BtFAD2‐9 (Figure S3A, Supporting Information).

### 2.2 Expression Profiling and Phylogenetic Analysis of *BtFAD2* Genes

The expression pattern of *BtFAD2* genes was monitored with real‐time quantitative PCR (qPCR) for all developmental stages (eggs, 1st–2nd, 3rd, 4th instar nymphs, female and male adults) of *B. tabaci* MED. The expression was particularly high in adults, especially males, suggesting that these genes play important roles at this stage. Most of the genes were differentially expressed in male and female adults, which implies they have crucial sex‐biased functions in *B. tabaci* (Figure 1B). Importantly, our initial transcriptome data from male and female adults indicated that the expression of *BtFAD2‐9* was significantly higher than all the other *BtFAD2* genes (Figure S3B, Supporting Information).

Our Bayesian phylogenetic analysis showed that all BtFAD2 proteins, as well as FAD2 proteins from other Aleyrodinae insects, *Trialeurodes vaporariorum*, *Aleyrodes proletella*, *Dialeurodes citri*, *Aleurocanthus spiniferus* and *Aleuroclava psidii*, clustered together with plant FAD2 proteins, while other functional insect Δ12 desaturases form a separate clade (Figure 1C; Figure S4, Supporting Information). Among all BtFAD2 proteins, BtFAD2‐9 clustered with BtFAD2‐10 had an ortholog in each Aleyrodinae species included in our analysis and was most closely related to plant FAD2s. The BtFAD2‐9 protein was highly similar to other *B. tabaci* cryptic species (MED_009496 in MED and Bta09295 in MEAM1) but also shared high protein similarity (61%−85%, except for the DcFAD2 partial protein) with other Aleyrodinae FAD2s (Figure S5 and S3A, Supporting Information). These results prompted us to further focus on the role of the *BtFAD2‐9* gene in *B. tabaci*.

### 2.3 Horizontal Transfer of *BtFAD2‐9* From Plants to Whiteflies

**Figure 2:**
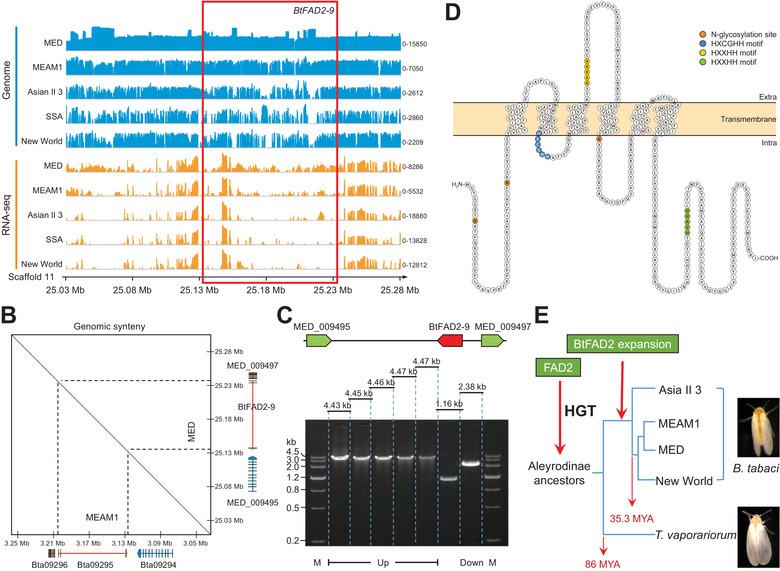
Horizontal transfer of *BtFAD2‐9* into *B. tabaci*. A) Genomic location of *BtFAD2‐9* gene in *B. tabaci* MED. Illumina DNA‐read coverage plots resulting from genomic sequencing of different *B. tabaci* cryptic species and Illumina RNA‐seq read coverage plots from diverse *B. tabaci* cryptic species adults are displayed. The sequence depths are denoted by the numbers on the right of the coverage plots. B) Genome synteny of the *BtFAD2‐9* gene and their respective two neighboring insect genes in *B. tabaci* MED (MED_009495 and MED_009497) and MEAM1 (Bta09294 and Bta09296). The black diagonal line indicates more than 95% similarity of two genomic regions. For *BtFAD2‐9*, red rectangles mean exons, red lines mean introns, and green rectangle means untranslated region. For two neighboring insect genes, orange and blue rectangles mean exons, orange and blue lines mean introns. C) Genome fragments cloned by overlapping PCR from *B. tabaci* MED. Genome fragment of *BtFAD2‐9* (MED_009496) with its upstream gene (MED_009495) and downstream gene (MED_009497). D) The structure of BtFAD2‐9 protein with five transmembrane domains generated by Protter. The N‐glycosylation site and the three histidine clusters are labeled in grey, blue, yellow, and green respectively. E) Diagram of the evolutionary history of FAD2 in Aleyrodinae insects. The event (86 MYA) when *Bemisia* divided from *Trialeurodes* and the event (35.3 MYA) when *B. tabaci* divided into different cryptic species are indicated. Abbreviation: Asia II 3: *B. tabaci* Asia II 3; New World: *B. tabaci* New World; MED: *B. tabaci* MED; MEAM1: *B. tabaci* MEAM1.

A BLAST search against the GenBank database revealed that except for homologs of *B. tabaci* MEAM1 (XP_018898615.1 in *B. tabaci* MEAM1), BtFAD2‐9 closest homologs were all plant proteins. Genomic analyses were performed to verify whether *BtFAD2‐9* was inserted into the genome of *B. tabaci* MED. Results showed that the *BtFAD2‐9* genomic region located at scaffold 11 was accurately assembled and highly consistent among different *B. tabaci* cryptic species (**Figure** **2**A). Genomic regions of the *BtFAD2‐9* gene and their surrounding genes of *B. tabaci* MED share highly conserved synteny with *B. tabaci* MEAM1 (Figure 2B). Furthermore, overlapping PCR amplicons of those genomic regions confirmed the assembling accuracy and ensured that *BtFAD2‐9* is indeed integrated into the *B. tabaci* MED genome (Figure 2C). Like plant FAD2 proteins, BtFAD2‐9 has six transmembrane domains (Figure 2D) and, similar to other Aleyrodinae FAD2 proteins, exhibits three typical histidine clusters (Figures S2C,D, Figures S6A,B, Supporting Information), which is distinct from the other functional insect Δ12 desaturases. Similar to a previous report,^[^ ^17^ ^]^ our phylogenetic analysis showed that *FAD2* genes were present before the split of the Aleyrodinae and, most likely, were not acquired independently by whitefly‐species (Figure 2E). Further, BtFAD2 gene duplication events seem to have occurred before the divergence of the *B. tabaci* cryptic species (≈35.3 MYA). Overall, our analyses show that the *BtFAD2‐9* gene is not a plant gene contaminant, but that Aleyrodinae ancestors must have horizontally acquired it from a host plant.

### 2.4 BtFAD2‐9 Protein Desaturates Oleic Acid to Produce Linoleic Acid

**Figure 3:**
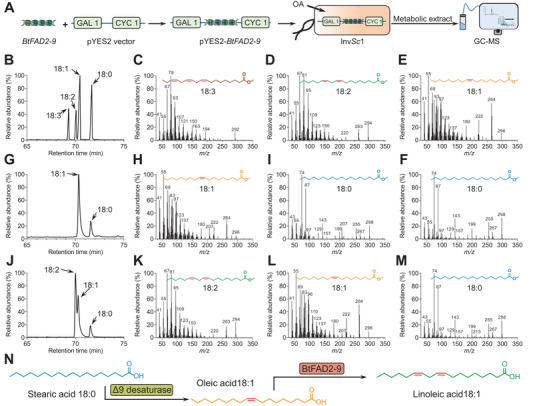
Metabolic analyses of BtFAD2‐9 enzyme activity. A) *BtFAD2‐9* was ligated to the pYES2 vector and transformed into yeast for heterologous expression. Yeast metabolites were extracted and analyzed using a GC‐MS system. B) Chromatograms of fatty acid methyl esters (FAME) standards (FAME of linolenic acid, 18:3^Δ9, 12, 15^; linoleic acid, 18:2^Δ9, 12^; oleic acid, 18:1^Δ9^; stearic acid, 18:0). C‐F) Secondary mass spectrometry chromatograms of FAME standards related to (B). G) Chromatograms of empty vector transgenic yeast metabolites (FAME of oleic acid, 18:1^Δ9^; stearic acid, 18:0). H‐I) Secondary mass spectrometry chromatograms of yeast metabolites related to (G). J) Chromatograms of *BtFAD2‐9* transgenic yeast metabolites (FAME of linoleic acid, 18:2^Δ9, 12^; oleic acid, 18:1^Δ9^; stearic acid, 18:0). K‐M) Secondary mass spectrometry chromatograms of yeast metabolites related to (J). N) Fatty acid catalytic processes in *BtFAD2‐9* transgenic yeast.

The plant‐derived *BtFAD2‐9* gene was heterologously expressed in *Saccharomyces cerevisiae* (**Figure** **3**A) to conduct in vitro enzyme activity assays. Fatty acid analysis of lipid extracts from yeast clones expressing the recombinant BtFAD2‐9 showed an additional peak of linoleic acid (C18:2Δ^9,12^) that was absent in the controls (Figure 3B–M). Further, we confirmed that the BtFAD2‐9 enzyme is a Δ12 fatty acid desaturase, converting oleic acid (C18:1Δ^9^) into linoleic acid (C18:2Δ^9,12^) (Figure 3N). Thus, BtFAD2‐9 completes the key desaturation steps required for de novo biosynthesis of PUFAs. In contrast, we did not detect α‐linolenic acid (C18:3Δ^9,12,15^) or γ‐linolenic acid (C18:3Δ^6,9,12^), suggesting that linoleic acid (C18:2Δ^9,12^) cannot serve as a substrate for the BtFAD2‐9 protein. Evidently, BtFAD2‐9 specifically catalyzes the synthesis of linoleic acid from oleic acid but has no catalytic activity for other fatty acids like linoleic acid.

### 2.5 Spatio‐temporal Expression Profiling of the *BtFAD2‐9* Gene

**Figure 4:**
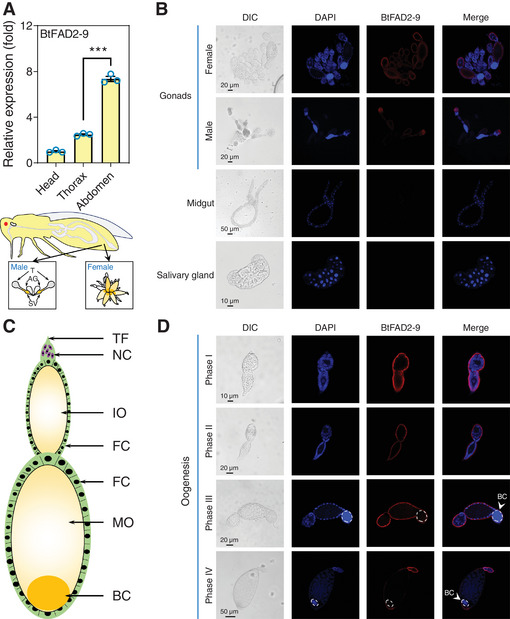
Localization of BtFAD2‐9 in different tissues of whitefly. A) Relative expression levels of *BtFAD2‐9* gene in the head, thorax, and abdomen of adult whitefly. The model below shows the structure and main organs of the whitefly. B) Immunofluorescence (IF) localization of the BtFAD2‐9 protein in different tissues of the whitefly using the rabbit polyclonal anti‐BtFAD2‐9 antibody. Nuclei are shown in blue, red is the positive signal for anti‐BtFAD2‐9. C) Structure of the ovariole of the whitefly. D) Localization of BtFAD2‐9 protein in follicular cells and oocytes of ovarioles at different developmental phases. Nuclei are stained with DAPI (blue), red is the positive signal for anti‐BtFAD2‐9. Abbreviations are as follows: T, testis; SV, seminal vesicle; AG, accessory gland; TF, terminal filament; NC, nurse cell; IO, immature oocyte; FC, follicle cell; MO, mature oocyte; BC, bacteriocyte. Data are presented as means ± SEM (A), *n* = 3 (A) biologically independent samples, *P < 0.05, **P < 0.01, ***P < 0.001, one‐way ANOVA with Tukey's test was used in (A) for comparison.

To further study the functional role of the *BtFAD2‐9* gene, its spatio‐temporal expression patterns were monitored by qPCR and immunofluorescence. qPCR analysis showed that *BtFAD2‐9* was expressed in various parts of *B. tabaci* adults (head, thorax, and abdomen). It is most highly expressed in the abdomen, which suggests that *BtFAD2‐9* mainly active its functions in this insect body part (**Figure** **4**A). Immunofluorescence showed that the BtFAD2‐9 protein is specifically located in the gonads and not in the midgut and salivary glands (Figure 4B). Because of the special structure of the insect's ovariole (Figure 4C), we examined the BtFAD2‐9 localization in different developmental stages of oogenesis in *B. tabaci*.^[^ ^20^ ^]^ The results showed that BtFAD2‐9 is expressed in all different stages of oogenesis, especially in phase I and phase II (Figure 4D). Moreover, the specific localization of BtFAD2‐9 in follicular cells suggests that BtFAD2‐9 plays an important role in follicular cells during oogenesis (Figure 4D). Together, these results indicate that BtFAD2‐9 is functional in the whitefly's gonads and highly expressed in its testes and follicular cells.

### 2.6 Functional Analysis of the *BtFAD2‐9* Gene Using RNAi and VIGS Assays

**Figure 5:**
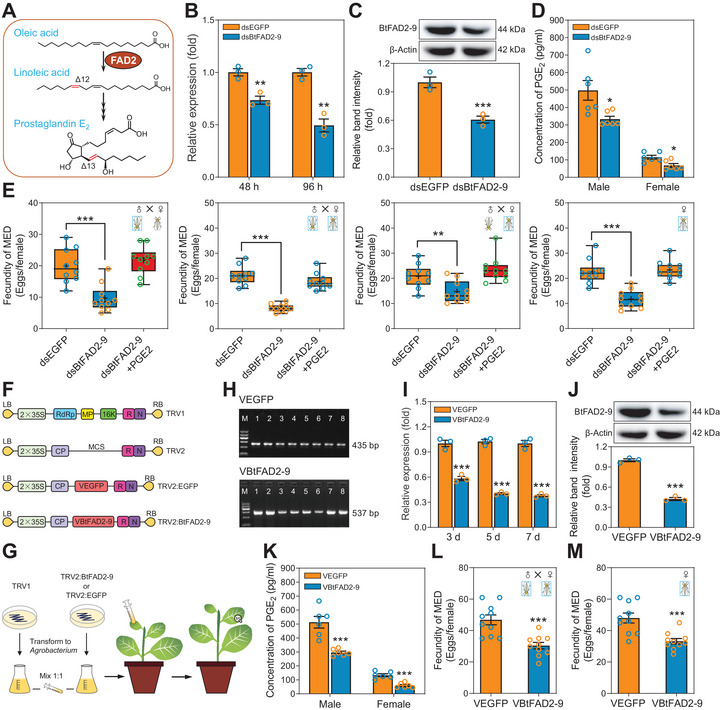
Effect of *BtFAD2‐9* silencing on *B. tabaci* performance. A) A proposed pathway from oleic acid to PGE_2_ and a role for *BtFAD2‐9* gene. Shown are proposed chemical structures based on GC‐MS data and click chemistry. B) The transcript levels of *BtFAD2‐9* at 48 and 96 h post‐RNAi as determined by qPCR. C) The relative expression levels of BtFAD2‐9 proteins at 96 h post‐RNAi. Both the detection of BtFAD2‐9 protein levels by Western blots (upper row) and quantitative estimation of band intensity by densitometry (graph) are presented. D) PGE2 concentration in *B. tabaci* adult male and female at 96 h post‐RNAi. E) Fecundity of *B. tabaci* in different mating groups. Whitefly adults were used in four treatments of mating: dsBtFAD2‐9 ♂ × control ♀; dsBtFAD2‐9 ♂ ×dsBtFAD2‐9 ♀; control ♂ ×dsBtFAD2‐9 ♀; dsBtFAD2‐9 ♀ for parthenogenesis. dsBtFAD2‐9 and dsBtFAD2‐9 plus PGE_2_ were set for each mating group. F) The constructed TRV‐based VIGS vectors. G) Procedure for persistent gene silencing of *B. tabaci* using TRV‐based vectors. H) PCR products amplified using cDNA from pTRV2‐EGFP (up) and pTRV2‐BtFAD2‐9 (down) tobacco leaves. M, marker (from top to bottom: 1,200 bp, 900 bp, 700 bp, 500 bp, 300 bp, 100 bp); lanes 1–10, PCR products (Up, 435 bp dsEGFP fragment; Down, 537 bp dsBtFAD2‐9 fragment). I) The transcript levels of *BtFAD2‐9* in *B. tabaci* adults feeding on VBtFAD2‐9 tobacco for 3, 5, and 7 days as determined by qPCR. J) The relative expression levels of BtFAD2‐9 proteins from *B. tabaci* adults feeding on VBtFAD2‐9 tobacco for 7 days. Both the detection of BtFAD2‐9 protein levels by Western blots (upper row) and quantitative estimation of band intensity by densitometry (graph) are presented. K) PGE2 concentration in *B. tabaci* adult male and female feeding on VBtFAD2‐9 tobacco for 7 days. L,M) Mating (L) and parthenogenesis (M) fecundity of *B. tabaci* feeding on VBtFAD2‐9 tobacco and VEGFP tobacco for 7 days. Values are means ± SEM, *n* = 3 (B, C, I and J), *n* = 6 (D and K), and *n* = 10 (E, L, M) biologically independent samples, *P < 0.05, **P < 0.01, ***P < 0.001, one‐way ANOVA with Tukey's test was used for comparison.

To further verify that *BtFAD2‐9* participates in PUFA biosynthesis (**Figure** **5**A), a series of in vivo RNA interference (RNAi) experiments were performed. Using tailored feeding capsules (Figure S7B, Supporting Information), *B. tabaci* MED adults were fed on gene‐specific dsRNA targeting *BtFAD2‐9*. qPCR analysis confirmed that the transcript and protein levels of *BtFAD2‐9* were both significantly decreased upon silencing for 96 h, while expression of other 12 *BtFAD2* genes did not change significantly (Figure 5B,C; Figure S7A, Supporting Information). Silencing *BtFAD2‐9* dramatically reduced the PGE_2_ content of whitefly males and females (Figure 5D). As PUFAs are ultimately converted to PGE_2_, these results substantiate the notion that *BtFAD2‐9* participates in PGE_2_ biosynthesis. Subsequent mating assays revealed that silencing of *BtFAD2‐9* not only remarkably decreased the fecundity of female adults but also affected the mating success of male adults (Figure 5E; Figure S7C, Supporting Information). Also, although silencing of *BtFAD2‐9* had no effect on egg hatchability, it resulted in a highly skewed sex ratio of the progeny (Figures S7D,E, Supporting Information). Importantly, after exogenous PGE_2_ supplementation in the artificial diet, the fecundity and offspring sex ratio of dsBtFAD2‐9‐fed whiteflies was restored (Figure 5E; Figure S7E, Supporting Information). Furthermore, to examine the effect of other *BtFAD2* genes on the fecundity of the whitefly, RNAi was also performed on another higher‐expressed gene, *BtFAD2‐2* (Figure S7F, Supporting Information). The results showed that silencing *BtFAD2‐2* has no significant effect on the whitefly's fecundity (Figure S7G, Supporting Information).

Subsequently, we tested the function of *BtFAD2‐9* in an ecologically relevant experiment using a virus‐induced gene silencing (VIGS) technique. We constructed VIGS vectors and infiltrated these into tobacco plants (Figure 5F,G). After two weeks, both silencing fragments of the *BtFAD2‐9* and *EGFP* genes were detected in the tobacco seedlings by PCR (Figure 5H). Next, *BtFAD2‐9* expression in *B. tabaci* adults that had been feeding on the tobacco seedlings for 7 days was assessed by qPCR. Relative to expression in adults feeding on pTRV2‐EGFP tobacco plants, *BtFAD2‐9* expression in adults feeding on pTRV2‐BtFAD2‐9 tobacco plants was reduced by 62.3% (Figure 5I). Assessment of protein levels confirmed that BtFAD2‐9 was reduced in whitefly samples after feeding on specific VIGS tobacco plants (Figure 5J). Continuous silencing of *BtFAD2‐9* by VIGS for 7 days significantly decreased PGE_2_ levels and reduced fecundity of *B. tabaci* male and female adults (Figure 5K–M). The VIGS experiment also confirmed that *BtFAD2‐9* gene affects the sex ratio but not egg hatchability (Figures S7H,I, Supporting Information).

### 2.7 Transgenic Expression of *BtFAD2‐9* in Drosophila

**Figure 6:**
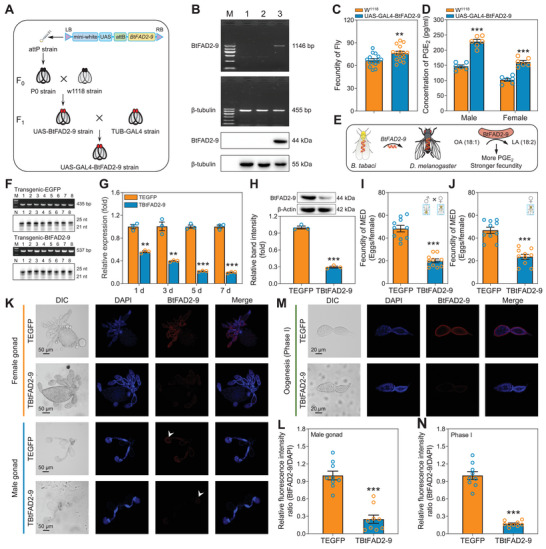
Transgenic fly and transgenic tobacco experiments show the effects of *BtFAD2‐9* on reproduction. A) Simplified schematic diagram of the construction of a transgenic *Drosophila melanogaster* strain. B) mRNA and protein of *BtFAD2‐9* in the transgenic fly lines. β‐tubulin was used as a reference gene. M, marker (Up‐PCR, from top to bottom: 1,200 bp, 900 bp, 700 bp, 500 bp, 300 bp, 100 bp; Down‐PCR, from top to bottom: 600 bp, 500 bp, 400 bp, 300 bp, 200 bp, 100 bp); lanes 1–3, PCR products or protein from W^1118^, UAS‐BtFAD2‐9 and UAS‐GAL4‐BtFAD2‐9 *D. melanogaster* line (from top to bottom: 1146 bp BtFAD2‐9 fragment, 455 bp β‐tubulin fragment, 44 kDa BtFAD2‐9 protein, 55 kDa β‐tubulin protein). C) Fecundity of W^1118^ and UAS‐GAL4‐BtFAD2‐9 *D. melanogaster* line. D) PGE2 concentration in male and female of W^1118^ and UAS‐GAL4‐BtFAD2‐9 *D. melanogaster* line. E) Functional model of *BtFAD2‐9* gene in transgenic *D. melanogaster* line. F) PCR and northern blot analyses from transgenic‐EGFP (up) and transgenic‐BtFAD2‐9 (down) tobacco leaves. For PCR gels: M, marker (from top to bottom: 900 bp, 700 bp, 500 bp, 300 bp); lanes 1–8, transgenic tobacco (Up, 435‐bp dsEGFP fragment; Down, 537‐bp dsBtFAD2‐9 fragment). For northern blot gels: N, negative control; lanes 1–8, transgenic tobacco. G) The transcript levels of *BtFAD2‐9* in *B. tabaci* adults feeding on transgenic‐BtFAD2‐9 tobacco for 1, 3, 5, and 7 days as determined by qPCR. H) The relative expression levels of BtFAD2‐9 proteins from *B. tabaci* adults feeding on transgenic‐BtFAD2‐9 tobacco for 7 days. Both the detection of BtFAD2‐9 protein levels by Western blots (upper row) and quantitative estimation of band intensity by densitometry (graph) are presented. I‐J) Mating (I) and parthenogenesis (J) fecundity of *B. tabaci* after feeding on transgenic‐BtFAD2‐9 tobacco and transgenic‐EGFP tobacco for 7 days. K‐L) Localization of BtFAD2‐9 protein in gonads of female and male whiteflies after feeding on transgenic‐BtFAD2‐9 tobacco and transgenic‐EGFP tobacco for 7 days. Nuclei are stained with DAPI (blue), and red is the positive signal for anti‐BtFAD2‐9 (K). The relative fluorescence intensity ratio of gonads of male whiteflies was quantified by ImageJ v.1.51 (L). M‐N) Localization of BtFAD2‐9 protein in oogenesis phase I of female whitefly after feeding on transgenic‐BtFAD2‐9 tobacco and transgenic‐EGFP tobacco for 7 days. Nuclei are stained with DAPI (blue), and red is the positive signal for anti‐BtFAD2‐9 (M). The relative fluorescence intensity ratio of oogenesis phase I of female whiteflies was quantified by ImageJ v.1.51 (N). Values are means ± SEM, *n* = 3 (G and H), *n* = 6 (D), *n* = 9 (L and N), *n* = 10 (I and J), and *n* = 15 (C) biologically independent samples, *P < 0.05, **P < 0.01, ***P < 0.001, one‐way ANOVA with Tukey's test was used for comparison.

To further confirm the metabolic function of *BtFAD2‐9*, we also ectopically expressed *BtFAD2‐9* into *Drosophila melanogaster*, which is a model insect that lacks the capacity for Δ12 desaturation, through the UAS‐GAL4 system (**Figure** **6**A). We confirmed the *BtFAD2‐9* gene was expressed in transgenic *D. melanogaster* by PCR and Western blot (Figure 6B). Second, to test the effect of the *BtFAD2‐9* gene on the fecundity of *D. melanogaster*, we recorded the egg production of transgenic and control flies. The results show that the transfer of the *BtFAD2‐9* gene enhanced egg production in *D. melanogaster* (Figure 6C). Moreover, higher levels of PGE_2_ were detected in the *D. melanogaster* expressing *BtFAD2‐9* line (UAS‐GAL4‐BtFAD2‐9) compared to the control line (W^1118^) (Figure 6D). Taken together, the transformation with the *BtFAD2‐9* gene enhanced the ability to synthesize PUFAs thereby allowing *D. melanogaster* to acquire more PGE_2_ and produce more eggs (Figure 6E).

### 2.8 Transgenic Expression of dsBtFAD2‐9 in Tobacco Decreases Fertility of Whiteflies

To investigate whether *BtFAD2‐9* contributes to the exceptional host adaptability of *B. tabaci*, the gene‐specific dsRNA expressed vector (pCAMBIA‐RNAi‐dsBtFAD2‐9) expressing hairpin RNA of *BtFAD2‐9* was constructed (Figure S8A, Supporting Information) and transferred into tobacco (Figure S8B, Supporting Information). Positive transgenic lines were identified by PCR amplification (Figure 6F). Northern blot analyses confirmed that the positive transgenic lines generated target small interfering RNAs (siRNAs) (Figure 6F). The transcript level of the *BtFAD2‐9* gene was significantly reduced after whiteflies had fed on such transgenic‐BtFAD2‐9 tobacco plants (Figure 6G), and assessments of protein levels confirmed that their BtFAD2‐9 content was markedly reduced (Figure 6H). Most importantly, feeding on transgenic plants significantly reduced the whiteflies’ capacity to reproduce, either parthenogenetically or sexually (Figure 6I,J). In a long‐term experiment, transgenic plants significantly altered the whitefly's sex ratio but not egg hatchability (Figures S8C,D, Supporting Information). In addition, immunofluorescence assays revealed a significant reduction in BtFAD2‐9 protein levels within the gonads of male and female whitefly adults feeding on the transgenic plants (Figure 6K,L). They also showed a significant reduction in BtFAD2‐9 during oogenesis, especially in the first phase (Figure 6M,N; Figure S9A–C, Supporting Information). These results show that *BtFAD2‐9* is of great importance for whitefly reproduction.

## 3 Discussion

PUFAs are indispensable nutrients for all living organisms. It has long been thought that most animals are incapable of de novo biosynthesizing PUFAs and that they can only acquire them through their diet. Unlike plants and certain microorganisms, animals mostly lack Δ12 desaturase, a key enzyme responsible for PUFA biosynthesis by desaturating MUFAs.^[^ ^8^ ^]^ However, some insects have been shown to synthesize PUFAs autonomously with their own Δ12 desaturases, which might have independently evolved from ancestral desaturases like Δ9 and Δ11 desaturases.^[^ ^10a,b,g^ ^]^ The whitefly *B. tabaci* is also able to produce PUFAs^[^ ^10c^ ^]^ and has acquired PUFA synthesis genes from plants via an HGT event,^[^ ^11^, ^17^ ^]^ which was further confirmed in our analysis (Figure S10, Supporting Information). It is increasingly evident that this uncommon evolutionary route has occurred quite frequently in whiteflies.^[^ ^14^, ^21^ ^]^ Our phylogenetic analysis revealed that the *BtFAD2* gene family and other Aleyrodinae *FAD2* genes are distantly related to other functional insect Δ12 desaturase genes (Figure 1C). The results further show that all the examined Aleyrodinae species have acquired plant‐derived *FAD2* genes, strongly indicating that they might be present in all Aleyrodinae species (Figure 1C) and that the transfer of *FAD2* most likely occurred in the whitefly ancestor before the divergence of *B. tabaci* and *T. vaporariorum* (> 86 MYA) (Figure 2E).

*FAD2* is a significant gene family in numerous plants and is expressed at all developmental stages. It participates in processes such as stress resistance and seed germination.^[^ ^19^ ^]^ The evolution and diversification of the *FAD2* gene family in plants is species‐specific, and the vast expansion is the result of gene duplication events.^[^ ^22^ ^]^ However, functionally redundant genes generated by duplication cannot be stably retained in the genome unless they undergo functional evolution, such as pseudogenization, neo‐, sub‐ functionalization, or both.^[^ ^23^ ^]^ Indeed, a number of plant *FAD2* genes are known to exhibit neofunctionalization complementary to the conserved function of Δ12 desaturases, for example, hydroxylation, conjugation, and acetylation.^[^ ^24^ ^]^ In our study, the *BtFAD2* gene family includes 13 transcribed genes that might have been generated via several tandem‐repeated gene duplications during whitefly genome evolution (Figure 1A; Figure S2A, Supporting Information), and thereby possibly underwent neofunctionalization accompanied by subfunctionalization, which is similar to the case of plant FAD2. Additional phylogenetic analysis revealed that BtFAD2‐9, and its Aleyrodinae orthologs, were most closely related to plant FAD2s. This suggests that the Aleyrodinae *FAD2‐9* gene might be the ancestral *FAD2* gene from plants and, based on the presence of *BtFAD2‐2* orthologs in several Aleyrodinae species, was subsequently duplicated in the whitefly ancestor. However, further analyses are needed to fully elucidate the complex evolutionary history of *BtFAD2‐9* and *BtFAD2‐2*. Nevertheless, the available data does suggest that multiple gene duplication events occurred before the divergence of the *B. tabaci* cryptic species (≈ 35.3 MYA).^[^ ^17^, ^25^ ^]^ Intriguingly, a previous study indicated that the decrease in gene expression after duplication can be beneficial by rebalancing gene dosage.^[^ ^23^ ^]^ We therefore speculate that a similar transcriptional regulatory mechanism might modulate the differential expression of these tandem‐repeated *BtFAD2* genes to optimize their functions, which warrants further study.

We found that heterologous expression of the *BtFAD2‐9* gene in yeast catalyzes Δ12 desaturation of oleic acid to produce linoleic acid (Figure 3N), which matches with a previous finding that the whitefly synthesizes linoleic acid from dietary radiolabeled acetate.^[^ ^10c^ ^]^ Our results further show that silencing the *BtFAD2‐9* gene reduced the level of PGE_2_, the major prostaglandin involved in various undesirable metabolic anomalies.^[^ ^26^ ^]^ PGE_2_ is synthesized from PUFAs as substrate and is known to be involved in mammalian fertility, regulating oviduct ciliogenesis, contractility, and other reproductive processes.^[^ ^27^ ^]^ We found that silencing of the *BtFAD2‐9* gene in the whitefly reduced fecundity (Figure 5), while the transfer of *BtFAD2‐9* into *D. melanogaster* also enhanced its egg production (Figure 6C). In both cases, fecundity was positively correlated with the insects’ PGE_2_ levels_._ Indeed, *BtFAD2‐9* silencing in whiteflies could be rescued by supplementing their diet with PGE_2_ (Figure 5E), and therefore, akin to their role in mammals, the *BtFAD2‐9* gene most likely affects whitefly fecundity by contributing to the production of prostaglandins. Indeed, levels of the BtFAD2‐9 protein are particularly high in the follicular cells of the female ovariole, which is an important composition for oogenesis.^[^ ^28^ ^]^ Follicular cells provide protection to the oocyte, are involved in material transportation during oogenesis, and serve as precursor tissue for eggshell development in insects.^[^ ^29^ ^]^ The BtFAD2‐9 protein was also found to be highly concentrated in the testis of whitefly males, where sperm development takes place,^[^ ^30^ ^]^ implying that BtFAD2‐9 is also involved in the reproductive system of whitefly males. In insects, males deliver prostaglandins to females through mating, which facilitates egg fertilization and promotes egg‐laying behavior.^[^ ^31^ ^]^ However, our experiments showed that silencing of the *BtFAD2‐9* gene also reduces the fecundity of whitefly females that reproduce asexually through parthenogenesis. This implies that asexually reproducing whitefly females, also produce prostaglandins themselves with the use of *FAD2*, without having to rely on their diet or males. Altogether, it is clear from our study that BtFAD2‐9 is involved in the synthesis of PGE_2_ in gonad cells to promote the sexual reproductive process of whiteflies, but further studies are needed to unravel its precise role in parthenogenesis.

**Figure 7:**
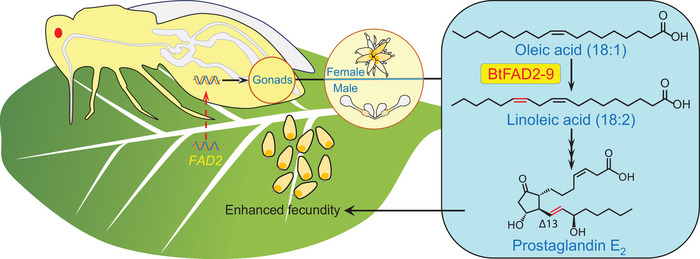
Schematic overview of how the acquisition of the plant gene *BtFAD2‐9* empowers the whitefly *B. tabaci* to enhance its fecundity. In *B. tabaci*, *BtFAD2‐9* gene is highly expressed in the gonads. BtFAD2‐9 can use oleic acid as a substrate to catalyze the synthesis of linoleic acid, which is eventually used to synthesize prostaglandins E_2_ for reproduction. With this ability, *B. tabaci* adults can synthesize PUFA by themselves, enhancing their reproductive output, which may have contributed to their adaptability to a large range of host plants.

Host plant nutrient quality is a key determinant of the fecundity of herbivorous insects.^[^ ^32^ ^]^ Our findings suggest that *B. tabaci* can reduce this host plant dependency thanks to the horizontally transferred *FAD2* gene (**Figure** **7**). This echoes the Chinese proverb, “give a man a fish and you feed him for a day; teach a man to fish and you feed him for a lifetime”. In this context, it is increasingly evident that HGT is an impetus for biological evolution and genetic innovation, and has provided recipient organisms with highly efficient control over biological processes.^[^ ^33^ ^]^ For example, several horizontally transferred essential amino acid biosynthesis‐related genes have been identified in *B. tabaci*,^[^ ^34^ ^]^ whereas the whitefly uses the horizontally transferred genes *BioA*, *BioB*, and *BioD* to compensate for the lack of biotin synthesis, again reducing their dependency on endosymbionts.^[^ ^35^ ^]^ Whiteflies, as piercing‐sucking pests, feed mainly on the phloem sap of plants, which usually contains only trace amounts of fatty acids.^[^ ^36^ ^]^ Hence, the horizontally transferred *FAD2* gene compensates for *B. tabaci*’s limited access to fatty acid nutrients and might have a similar function in all Aleyrodinae species.

Silencing of the *BtFAD2‐9* gene results in a significant decrease in the fecundity of *B. tabaci* (Figure 5E), which greatly reduces insect performance and could be exploited for crop protection. Hence, this study not only provides insight into a co‐evolutionary process that facilitates nutrient acquisition in insects but also reveals that interfering with laterally transferred genes could be a highly effective way to combat pests.^[^ ^14^, ^37^ ^]^ Targeting the reproductive process of insects is an important aspect of pest control and currently is applied in various pest management strategies.^[^ ^38^ ^]^ One of the most popular methods of inhibiting pest fecundity is via *Wolbachia*, an endosymbiotic bacterium that occurs in a broad range of invertebrates. *Wolbachia* reduces the fecundity of pests by inhibiting mating success.^[^ ^39^ ^]^ However, this method is less effective against pests like whiteflies that can reproduce through parthenogenesis.^[^ ^40^ ^]^ We show that silencing *BtFAD2‐9* significantly reduces the parthenogenetic fertility of the whitefly, thus providing a mating‐independent strategy for pest control. A thorough screening for other such fertility‐related genes might yield excellent targets for the promising RNAi‐based insect pest control strategy.

To summarize, this study illustrates that important physiological traits do not necessarily originate from the evolution of endogenous pre‐existing genes, but can be acquired by exogenous HGT events, highlighting an alternative pathway of evolution. The ability of de novo PUFA biosynthesis has important ecological consequences because it implies that whiteflies are released from a dependency on dietary PUFAs. PUFAs serve important functions in all organisms, such as energy storage, mobilization, and transport, as well as structural components in membranes. They also have a number of functions that are apparently more‐or‐less unique to insects and have the potential to become emerging areas of interest in insect biochemistry and physiology. Indeed, there is growing evidence that integrative studies on insect PUFAs can reveal important principles of animal metabolism, including mechanisms of PUFA biosynthesis, and causes of metabolic diseases like obesity and cancer.^[^ ^41^ ^]^

## 4 Experimental Section

### Insect Strain

A cotton strain of *B. tabaci* MED was created from individuals initially collected from poinsettia (*Euphorbia pulcherrima* Wild. ex Klotz.) plants in Beijing, China in 2009, that were then transferred to cotton (*Gossypium herbaceum* L. cv. DP99B) plants. A tobacco strain of *B. tabaci* MED was created in 2017 from the above parental cotton strain, by rearing it continuously on tobacco (*Nicotiana tabacum* K326).^[^ ^42^ ^]^ The purity of the *B. tabaci* MED strain was monitored by sequencing a fragment of the mitochondrial cytochrome oxidase I (mtCOI) gene every three to five generations.^[^ ^43^ ^]^ All the experiments in this study were conducted using the tobacco strain, which was maintained in a glasshouse at 27 ± 1 °C, 60%–80% relative humidity (RH), and a photoperiod of 14 h light/10 h darkness.

### RNA Isolation and cDNA Synthesis

Total RNAs were extracted from various whitefly samples using the TRIzol reagent (TaKaRa) according to the manufacturer's recommendations. Agarose gel electrophoresis was used to determine the integrity of the RNA, and NanoDrop 2000c spectrophotometer (Thermo Fisher Scientific) detection was used to quantify the RNA. cDNAs were synthesized using the PrimeScript II 1st Strand cDNA Synthesis Kit (TaKaRa) and the PrimeScript RT Kit (containing gDNA Eraser, Perfect Real Time) (TaKaRa) for *BtFAD2* gene cloning and qPCR analysis, respectively. The synthesized cDNAs were immediately stored at −20 °C until used.

### Gene Identification and Cloning

The *BtFAD2* genes were originally found in our previously sequenced *B. tabaci* MED genome (https://www.gigadb.org/dataset/100286) and transcriptome libraries,^[^ ^44^ ^]^ and they were re‐evaluated by BLASTp against the GenBank database (https://www.ncbi.nlm.nih.gov/). The putative coding sequences (CDSs) of these *BtFAD2* genes were manually corrected using the previously completed transcriptome data of *B. tabaci* MED.^[^ ^45^ ^]^ Specific primers used for gene cloning (Table S1, Supporting Information) were designed using Primer Premier 5.0 (https://www.premierbiosoft.com/primerdesign/). The PCR reactions were conducted using LA Taq polymerase with high GC buffer (TaKaRa). The detailed programs of the PCR analyses are listed below: denaturing at 94 °C for 10 min; cycling 35 times with the following parameters: denaturing at 94 °C for 60 s, annealing at 60 °C for 60 s, and extension at 72 °C for 2 min; final extension at 72 °C for 10 min. The obtained amplicons of *BtFAD2* were purified, cloned into the pEASY‐T1 vector (TransGen), and sequenced, and the finally obtained full‐length cDNA sequences of all the MED *BtFAD2* genes have been deposited in the GenBank database (accession nos. OQ291260‐OQ291272).

### Phylogenetic Analysis

For phylogenetic tree construction, the protein sequences of *BtFAD2* genes were used as queries in a BLASTp (with “Expect threshold” set at 1E‐15) search against the NCBI‐non‐redundant protein database to identify homologs. For the top 30 hits in each BLASTp result, we downloaded the complete protein sequence of the representative hits. De novo transcriptomes were assembled for three Aleyrodinae species – *Aleyrodes proletella*, *Dialeurodes citri*, and *Aleurocanthus spiniferus* while a de novo genome was generated for the Aleyrodinae whitefly *Aleuroclava psidii*, using CLC Genomics Workbench version 22.0.1 with default settings and previously deposited sequencing data (Sequence Read Archives: SRR18920704/SRR18920710/SRR18920713/SRR18920719/SRR18920722/SRR18920723, SRR949617/SRR1015076, SRR17330021‐26 and SRR16114381, respectively). BtFAD2 proteins were used in a tBLASTn search against these transcriptomes, the *A. psidii* de novo genome, and the previously published *Trialeurodes vaporariorum* gene annotation.^[^ ^46^ ^]^ Those tBLASTn hits were extracted with an 80% identity threshold to avoid picking up contaminating plant FAD2s, and named them as AprFAD2‐1, AprFAD2‐2, DcFAD2‐1, DcFAD2‐2, AsFAD2‐1, AsFAD2‐2, ApsFAD2, and TvFAD2, respectively (Table S3, Supporting Information). Further, only sequences [AdD12Des (*Acheta domesticus*, ABY26957.1), ClD12Des (*Chauliognathus lugubris*, AFJ66832.1), NvD12Des1 (*Nasonia vitripennis*, XP 001599836.1) and NvD12Des2 (XP 001599873.1) were added, TcD12Des (*Tribolium castaneum*, NP 001137206.1)] that were reported to have active Δ12 desaturation functions in insects for phylogenetic analysis. Other insect homologs annotated as potential Δ9 or Δ11 desaturase were excluded to make the constructed phylogenetic tree more accurate. The sequence set was filtered for redundancy and protein length (>250 aa), resulting in a final dataset of 238 sequences (Table S2, Supporting Information). These sequences were aligned with MAFFT v7.311 using L‐INS‐I option (https://www.mafft.cbrc.jp/alignment/software). Before constructing the phylogenetic trees, the best protein substitution matrixes (JTT + I + G protein substitution matrix at 859 aligned amino acid positions) were predicted by Prottest V3.4 (https://github.com/ddarriba/prottest3). The alignment was used to infer the phylogenetic tree by MrBayes V3.2.7a (https://nbisweden.github.io/MrBayes/). MrBayes was run with two Markov chains over 50 000 000 generations with sampling frequency of 1 tree for every 1000 generations. The Bayesian posterior probabilities of trees, after discarding the first 25% of trees as burn‐in, were calculated with the remaining trees. The resulting tree was converted into a Newick format using AfterPhylo V0.9.1 (https://github.com/qiyunzhu/AfterPhylo). For phylogenetic analysis of the *BtFAD2* family, MrBayes was run with the best protein substitution matrixes (WAG + G protein substitution matrix at 700 aligned amino acid positions) and the above parameters. All the phylogenetic trees were midpoint‐rooted and annotated by iTOL (https://itol.embl.de/).

### Bioinformatic Analysis

A bioinformatics approach was used to confirm the incorporation of *BtFAD2‐9* in *B. tabaci* MED genome as previously reported.^[^ ^14^ ^]^ In brief, paired‐end Illumina genomic sequencing reads of *B. tabaci* MED, *B. tabaci* MEAM1, *B. tabaci* Asian II3, *B. tabaci* SSA, *B. tabaci* New World were respectively mapped to the *B. tabaci* MED genome with Bowtie2 2.4.0 with default parameters (https://www.bowtie‐bio.sourceforge.net/bowtie2). Paired‐end Illumina transcriptome sequencing reads of *B. tabaci* MED, *B. tabaci* MEAM1, *B. tabaci* Asian II3, *and B. tabaci* SSA were also mapped to the genomic region of the *B. tabaci* MED with STAR 2.7.0b with default parameters (https://github.com/alexdobin/STAR). Alignments were sorted with samtools 1.17 with default parameters (https://github.com/samtools/) and visualized with IGV 2.16.0 (https://software.broadinstitute.org/software/igv/). Coverage of each alignment was also calculated by IGV. The protein transmembrane helix was predicted by the online server Protter (https://wlab.ethz.ch/protter/start/).

### Genomic DNA Isolation and Cloning

A total number of 100 *B. tabaci* MED adults was collected and ground into powder with liquid nitrogen. Genomic DNA (gDNA) was isolated using the TIANamp Genomic DNA Kit (TIANGEN) following the manufacturer's instructions. The DNA purity was evaluated by 1% agarose gel electrophoresis and the DNA concentration was measured using a NanoDrop2000c spectrophotometer (Thermo Fisher Scientific). Based on the *B. tabaci* MED genome sequences, specific primers were designed by Primer Premier 5.0 to amplify the intergenic genomic regions of *BtFAD2‐9*. Amplicons of each PCR were purified, cloned into the pEASY‐T1 vector, and sequenced (Tsingke).

### qPCR Analysis

The expression levels of target genes were quantified using the QuantStudio 3 Real‐Time PCR System (Applied Biosystems). The gene‐specific primers of *BtFAD2* used for the real‐time quantitative PCR (qPCR) analysis were designed by the Primer Premier 5.0 software (Table S1, Supporting Information). The 25 µL PCR reactions included 0.5 µL of 50 × ROX Reference Dye (TIANGEN), 0.75 µL of each specific primer, 1 µL of cDNA template, 9.5 µL of ddH_2_O, and 12.5 µL of 2 × SuperReal PreMix Plus (SYBR Green) (TIANGEN). The qPCR reactions were performed in an ABI 7500 system (Applied Biosystems) with the following protocol: initial denaturation of 94 °C for 3 min, followed by 40 cycles of 95 °C for 15 s, 60 °C for 30 s, and 72 °C for 30 s. The amplification efficiencies were determined by dissociation curve analysis using five two‐fold serial dilutions of *B. tabaci* cDNA template. Only primers with 90%–110% amplification efficiencies were used for the subsequent studies.

Relative quantification was calculated according to the 2^−ΔΔCt^ method,^[^ ^47^ ^]^ to accurately analyze the expression of the target genes, the expression data were normalized to the internal gene elongation factor 1 alpha (*EF1‐a*) (GenBank accession number EE600682). Three independent biological replicates and four technical replicates were performed for each whitefly sample.

### Yeast Transformation and Enzyme Activity Assays

The function of *BtFAD2‐9* was in vitro characterized in the yeast *Saccharomyces cerevisiae* system. Briefly, the predicted open reading frame (ORF) of *BtFAD2‐9* gene was amplified by PCR using primers containing restriction enzyme sites (HindIII and EcoRI) for further cloning into the yeast expression vector pYES2 (Invitrogen) (Table S1, Supporting Information). The pYES2 construct containing *BtFAD2‐9* ORF was sequenced prior to being used to transform the InvSc1 yeast line (Invitrogen). Yeast transformed with the pYES2 vector was cultured overnight in 2% raffinose, 1% Nonidet P‐40, and SC‐U medium (uracil dropout medium) at 30 °C with shaking. The cultures were then grown to an OD_600_ of 1 and 2% galactose (wt/vol) was added to induce transgene expression. Transgenic yeast expressing *BtFAD2‐9* were grown in the presence of exogenously added oleic acid substrates (25 mmol L^−1^). Control treatments consisted of yeast transformed with the empty pYES2 and run under the exact same conditions as above. After galactose induction for 48 h, equal amounts of yeast cultures were collected by centrifugation and dried under a stream of oxygen‐free nitrogen. To prepare yeast fatty acid methyl ester (FAME), the dried yeast cells were incubated with 2 mL 0.4 mol L^−1^ potassium hydroxide/methanol solution for 30 min at 37 °C with vortex shaking. Subsequently, 1 mL of 0.9% NaCl and 1 mL of hexane were added for 10 min at 37 °C with vortex shaking. Ultimately, the top phase was collected after phase separation for subsequent assays. Metabolic functions of the *BtFAD2‐9* were established by comparing the fatty acid profiles of *BtFAD2‐9* transformed yeast with those of the controls.

### GC‐MS Analysis

The fatty acid composition was analyzed by the coupled gas chromatography‐mass spectrometry (GC‐MS). The samples were dried and then dissolved in n‐hexane: toluene (1:1) prior to analysis on an Agilent GC‐MS instrument (7890B‐5977A, Agilent) with an HP‐5 MS columns (30 m × 0.25 mm inner diameter, film thickness, 0.25 µm, Agilent) and helium as the carrier gas. GC was performed with temperature‐programmed automatic injection at 60 °C, holding for 5 min at 60 °C, temperature increase to 230 °C at a rate of 2 °C min^−1^, and holding for 40 min at 230 °C. The identity of the desaturation products was determined by comparing their retention times with FAME contained in commercial standards (Sigma‐Aldrich, 18919‐1AMP).

### Western Blots

The antibody of *BtFAD2‐9* protein used for Western blots was generated from synthetic peptides (Pujian Biotech) derived from respective specific amino acid sequences ^316^HHLFPTMPHYHAVEAC^330^, and other specific antibodies targeting β‐actin and β‐tubulin were commercially purchased (Abcam, ab115777 and ab18207). The protein level of target proteins was determined with Western blots using β‐actin or β‐tubulin as internal controls. The protein samples (*ca* 30 µg protein extracted from whitefly and *Drosophila* mixed adult simple, respectively) were isolated using 10% SDS‐PAGE and transferred onto PVDF membranes (Merck Millipore). The PVDF membranes were then blocked with blocking buffer containing BSA (CWBIO) at 25 °C for 1 h and incubated with the appropriate primary antibody (1:5000) at 4 °C overnight, followed by incubation with goat anti‐rabbit horseradish peroxidase‐conjugated secondary antibody (1:5000, CWBIO). The protein bands were visualized using the SuperSignal West Pico Chemiluminescent Substrate (Thermo Fisher Scientific), and the images were captured by the Tanon‐5200 Chemiluminescent Imaging System (Tanon). Densitometric analysis of the protein bands was performed using ImageJ v.1.51 software (https://www.rsbweb.nih.gov/ij/), and the relative band intensities were calculated based on densitometric ratios between target proteins and internal controls.

### Immunostaining Assay

Midguts, male and female gonads, and salivary glands were dissected from whitefly adults. The specimens were fixed in 4% paraformaldehyde for 1 h at room temperature and washed in phosphate‐buffered saline with 0.05% Tween‐20 (PBST) three times. Then, the samples were blocked in PBST containing 1% bovine serum albumin (BSA) (Solarbio) for 3 h at room temperature, followed by incubation with the appropriate *BtFAD2‐9* primary antibody (1:3000) at 4 °C overnight. Following rinsing five times in PBST, samples were incubated with goat anti‐rabbit IgG conjugated to Alexa 555 (Abcam, 1:200) as a secondary antibody for 1 h at room temperature. The samples were again rinsed five times in PBST, and mounted with Fluoroshield Mounting Medium with DAPI (Abcam). Sections were imaged using a Zeiss LSM710 confocal microscope using wavelength (DAPI: excitation at 353 nm, emission at 465 nm; BtFAD2‐9: excitation at 545 nm, emission at 572 nm) and 10× objective with additional zooming. Relative fluorescence intensity analysis of the protein bands was performed using the ImageJ v.1.51 software (https://imagej.nih.gov/ij/) by densitometry.

### dsRNA Synthesis and RNAi Assays

To confirm the role of *BtFAD2‐9* in the metabolism of fatty acids, the expression of *BtFAD2‐9* was knocked down by oral delivery of dsRNA to *B. tabaci* MED adults. Gene‐specific dsRNA primers for *BtFAD2‐9* (dsBtFAD2‐9, 537 bp) and *EGFP* (GenBank accession no: KC896843) (dsEGFP, 435 bp) containing a T7 promoter sequence were designed by the Primer Premier 5.0 software (Table S1, Supporting Information). To avoid potential off‐target effects, a BLASTn search (E‐value threshold with 1E‐05) of the designed dsRNA fragment in the GenBank (https://www.ncbi.nlm.nih.gov/) and *B. tabaci* genome databases (https://www.gigadb.org/dataset/100286) was done, and no hits to any other homologous genes were detected except for one plant gene (*Erythranthe guttatus*, XM_012974661.1), which further validated the specificity of the selected dsRNA fragment. The dsRNA of each gene was synthesized with T7 RiboMAX Express RNAi system (Promega) according to the manufacturer's instructions.

RNAi was performed with the above‐described artificial‐feeding system (Figure S7A, Supporting Information), using a diet solution containing dsRNA added between the two layers of Parafilm (200 µL of diet solution with 0.5 µg µL^−1^ dsRNA). The dsEGFP was synthesized and used as the negative control. RNAi efficacy was assessed by qPCR after the newly emerged *B. tabaci* adults had been fed for 48 and 96 h. In addition, the effect of silencing *BtFAD2‐9* on the other 12 *BtFAD2* gene expression levels was examined by qPCR.

To measure the effect of knockdown of *BtFAD2‐9* on whitefly fecundity, 50 male and 50 female newly emerged adult whiteflies were fed diet solution supplemented with dsEGFP, dsBtFAD2‐9, or dsBtFAD2‐9 supplemented with 500 pg µL^−1^ PGE_2_ for 96 h. Subsequently, five pairs of whiteflies in four treatment groups of mating: 1) dsRNA ♀ × dsRNA ♂, where a dsRNA‐feeding female mated with a dsRNA‐feeding male, 2) control ♀ × dsRNA ♂, where control female mated with dsRNA‐feeding male, 3) dsRNA ♀ × control ♂, where dsRNA‐feeding female mated with control male, 4) dsRNA ♀, dsRNA‐feeding females of the parthenogenesis group (Figure. S7A, Supporting Information). Whiteflies of these treatments were released into a clip‐cage attached to a tobacco plant and allowed to lay eggs, with ten replicates for each treatment. After 3 days, egg numbers were recorded. The offspring of these mating groups were reared for 35 days, during which period egg hatchability and adult sex ratio were determined. The effects of silencing *BtFAD2‐2* (Table S1, Supporting Information) were also examined on whitefly reproduction as described above. During RNAi experiments, the tubes and tobacco plants were placed in an MLR‐352H environmental chamber (Panasonic) at 25 °C and with a photoperiod of L14: D10 and 80% RH.

### PGE_2_ Measurement

*B. tabaci* or *D. melanogaster* samples were homogenized in the PBS buffer containing 1 mM ethylene diamine tetra acetic acid (EDTA) and 10 µM indomethacin. The mixtures were then centrifuged at 10 000 × *g* for 15 min at 4  °C to obtain supernatants. The concentrations of PGE_2_ in the supernatants were finally measured using the PGE_2_ ELISA Kit (MLBio) according to the manufacturer's instructions.

### VIGS Assays

To determine the effect of continuous interference with the *BtFAD2‐9* gene on *B. tabaci* reproduction and performance, virus‐induced gene silencing (VIGS) assays were carried out, and oviposition was assessed. Virus vectors for VIGS (pTRV1 and pTRV2) (Figure 5F) have been described previously.^[^ ^14^ ^]^ The experimental protocol is shown (Figure 5G). A *BtFAD2‐9* gene fragment was cloned from *B. tabaci* MED using specific primers (Table S1, Supporting Information), and the PCR product was then cloned into EcoRI‐BamHI‐cut pTRV2 to construct pTRV2‐BtFAD2‐9. A 435‐bp fragment of the *EGFP* gene was cloned using specific primers (Table S1, Supporting Information), and the PCR product was then cloned into EcoRI‐BamHI‐cut pTRV2 to construct pTRV2‐EGFP. The pTRV1, pTRV2‐BtBtFAD2‐9, and pTRV2‐EGFP vectors were transferred into *Agrobacterium tumefaciens* GV3101 by electroporation, and the bacteria were selected on LB agar plates containing 100 µg mL^−1^ of rifampicin and 50 µg mL^−1^ of kanamycin. The *A. tumefaciens* harboring pTRV1, pTRV2‐BtFAD2‐9 and pTRV2‐EGFP were validated by PCR amplification. The *A. tumefaciens* containing pTRV1 and pTRV2 carrying the target gene were added to 5 mL of liquid LB medium containing 100 µg mL^−1^ of rifampicin and 50 µg mL^−1^ of rifampicin and kanamycin; the cultures were kept for 18 h at 28 °C while shaking them at 200 rpm. A 2 mL volume of each culture was then added to 48 mL flesh liquid LB medium (100 µg mL^−1^ rifampicin, 50 µg mL^−1^ kanamycin, 200 µM acetosyringone, and 10 mM 2‐morpholinoethanesulfonic acid, MES); the cultures were once again kept for 18 h at 30 °C while shaking at 200 rpm. The cultures were then centrifuged at 3000 × *g* for 10 min, and the pellets containing *A. tumefaciens* were resuspended in 5 mL of infiltration medium (200 mM acetosyringone, 10 mM MES, and 10 mM MgCl_2_). The suspension was centrifuged again at 3000 × *g* for 10 min, and the pellets containing *A. tumefaciens* were resuspended in infiltration medium to obtain a final OD_600_ of 0.4 *A. tumefaciens* containing pTRV1 and pTRV2 with the target gene were mixed at a ratio 1:1. The mixture was infiltrated into the two largest true leaves of tobacco plants (*N. tabacum* K326) using a 1 mL needleless syringe, tobacco plants were left covered overnight. The infiltrated tobacco plants were kept in a growth chamber at 27 ± 1 °C, 70 ± 10% RH, and a photoperiod of L16: D8. After 20 days, total RNAs were extracted from the leaves of tobacco plants, and the cDNA samples were synthesized. With VIGS‐specific primers (Table S1, Supporting Information) and a tobacco cDNA template, PCR was used to determine whether the VIGS vectors successfully infected the tobacco host. The successfully infected tobacco plants (BtFAD2‐9‐VIGS tobacco and EGFP‐VIGS tobacco) were used for feeding assays.

To assess the effect of VIGS on whitefly performance, a clip cage harboring 60 newly emerged adults was placed on a nascent leaf of the selected tobacco plants. After feeding for 3, 5, and 7 days, the adult whiteflies were collected and their expression levels of *BtFAD2‐9* gene were measured. Then, five pairs of newly emerged adults (male and female) or 5 newly emerged female adults of *B. tabaci* MED were placed in each cage. Seven days later, the number of eggs laid on the leaf within the clip cages was counted. The offspring were reared for 35 days, during which egg hatchability and adult sex ratio were determined. In all the assays, EGFP‐VIGS tobacco plants were used as controls.

### Transgenic Flies

The ORF of *BtFAD2‐9* gene was amplified for further cloning into the p10 plasmid (pJFRC‐28‐10‐10 × UAS‐IVS‐GFP‐P10, Addgene plasmid # 36431). For phiC31 integrase‐mediated transformation on chromosome 3, p10‐BtFAD2‐9 plasmids were injected into attP40 *D. melanogaster* embryos by custom injection service provided by Qidong Fungene Biotechnology (Jiangsu Province, China) to generate transformant *UAS‐BtFAD2‐9* fly line for further crossings. *UAS‐BtFAD2‐9* flies were crossed with TUB‐GAL4 flies (BDSC # 57591) to generate the UAS‐GAL4‐BtFAD2‐9 line, and the homozygotes were obtained by sibling crosses. The fly adults were collected for the detection of *BtFAD2‐9* expression by PCR and Western blot. *D. melanogaster* β‐tubulin (GenBank accession no: NM_166356.2) was used as a reference gene. The primer sets are listed in Table S1 (Supporting Information). All fly lines were maintained at 25 ± 1 °C, 60% relative humidity, and 16:8 h light: dark photoperiod.

To detect the effect of heterologous expression of the *BtFAD2‐9* gene on *D. melanogaster* fecundity, a 5‐day‐old male and a virgin female were placed in a test tube for mating. The mated females were kept in food vials (1.06% agar, 3.22% yeast extract, 3.16% brown sugar, 6.32% glucose 7.74% cornmeal, and 1% nipagin), whereby fresh vials were provided every 3 days. The total number of adult progeny was counted in each vial. Fifteen independent assays were performed for each *D. melanogaster* sample. Finally, the concentrations of PGE_2_ in different *D. melanogaster* lines were measured as above.

### Transgenic Tobacco Plants

Transgenic tobacco lines were developed by introducing the hairpin RNA expression vector (pCAMBIA‐RNAi‐BtFAD2‐9) into tobacco (*N. tabacum* K326). The construction of a hairpin RNA expression vector (Figure S8A, Supporting Information) has been described previously.^[^ ^48^ ^]^ A 537‐bp target fragment of *BtFAD2‐9* was cloned from *B. tabaci* MED using sense‐BtFAD2‐9 primers (Table S1, Supporting Information), and the PCR product was then cloned into XhoI‐BglII‐cut pCAMBIA‐RNAi (pRNAi‐Sense‐BtFAD2‐9). The anti‐sense fragment of *BtFAD2‐9* was cloned from *B. tabaci* MED using anti‐sense‐BtFAD2‐9 primers (Table S1, Supporting Information), the purified product was then cloned into BamHI‐SalI‐cut pCAMBIA‐RNAi‐Sense‐BtFAD2‐9 (pRNAi‐BtBtFAD2‐9). *A. tumefaciens* LBA4404 based transformation was used to transfer the recombinant pCAMBIA‐RNAi‐BtFAD2‐9 plasmid into tobacco in a similar way as described previously for tomato.^[^ ^14^ ^]^ To verify the success of the transformation, gDNAs of putative transgenic tobacco leaves were extracted using the Plant Genomic DNA Kit (TIANGEN), and the extracted DNAs were subjected to PCR using detection primers (Table S1, Supporting Information). qPCR analyses were carried out to assess the RNAi efficacy on *B. tabaci* adults feeding on dsBtFAD2‐9 transgenic tobacco lines, *B. tabaci* adults feeding on the dsEGFP transgenic tobacco lines were used as a control. RNAi efficacy was determined every two days for seven days.

For determining the effects of *BtFAD2‐9* on *B. tabaci* reproduction, 5 pairs of newly emerged adults or five newly emerged female adults of *B. tabaci* MED were collected into one clip cage and fixed on the dsEGFP transgenic tobacco or dsBtFAD2‐9 transgenic tobacco plants. The newly laid whitefly eggs were recorded after 7 days. The offspring of these mating groups were reared to 35 days, to determine egg hatchability and adult sex ratio. Trans‐EGFP tobacco plants were used as controls.

### Northern blot

Northern blot analyses were performed to confirm the presence of the generated siRNAs in transgenic tobacco lines. The total RNAs of transgenic tobacco leaves were isolated and purified by TRIzol reagent (TaKaRa). Small RNAs were selectively recovered with 5% PEG8000 and 0.5 m NaCl from the purified total RNAs. The obtained small RNAs were then separated on denaturing 15% polyacrylamide gels and transferred onto Hybond‐N^+^ membranes (Amersham), and the membranes were further cross‐linked by exposure to UV light and hybridized to specific biotin‐labeled DNA probes that were generated by the PCR products labeled with Biotin‐dUTP (Beyotime). The results were visualized using the Chemiluminescent Biotin‐labeled Detection Kit (Beyotime), and the images were captured by the Tanon‐5200 Chemiluminescent Imaging System (Tanon).

### Statistical Analysis

All the data were analyzed using the IBM SPSS Statistics (ver. 23.0) software (IBM Corp.). Data are shown as the means ± SEM. The data's statistical significance was determined using one‐way ANOVA with Tukey's test (*, *P* < 0.05; **, *P* < 0.01; ***, *P* < 0.001).

## Conflict of Interest

The authors declare that a patent has been filed to the China National Intellectual Property Administration (application no. 202310263575.1).

## Author Contributions

C.G., Z.G., Y.H., and Z.Y. contributed equally to this work. C.G., Z.G., Z.Y. and Y.Z. designed the research. C.G., Z.G., Y.H., Z.Y., J.X., X.Y., W.X., S.W., and Q.W. performed the experiments. C.G. and Z.G. analyzed the data; C.G., Z.G., W.Y., X.Z., T.C.J.T., and Y.Z. wrote and revised the manuscript.

## Supporting information

Supporting Information

Supporting Information

## Data Availability

The data that support the findings of this study are available from the corresponding author upon reasonable request.
