# Supplementary material for: A Horizontally Transferred Plant Fatty Acid Desaturase Gene Steers Whitefly Reproduction
Source: Adv Sci (Weinh). 2023 Dec 25;11(10):2306653. doi: 10.1002/advs.202306653 (PMC10933598; doi:10.1002/advs.202306653)
Supplement: Supplementary file 1 — Supporting Information [file ADVS-11-2306653-s001.pdf]

ADVANCED SCIENCE
Open Access

Supporting Information

for *Adv. Sci.*, DOI 10.1002/advs.202306653

A Horizontally Transferred Plant Fatty Acid Desaturase Gene Steers Whitefly Reproduction

Cheng Gong, Zhaojiang Guo, Yuan Hu, Zezhong Yang, Jixing Xia, Xin Yang, Wen Xie, Shaoli Wang, Qingjun Wu, Wenfeng Ye, Xuguo Zhou, Ted C. J. Turlings and Youjun Zhang**

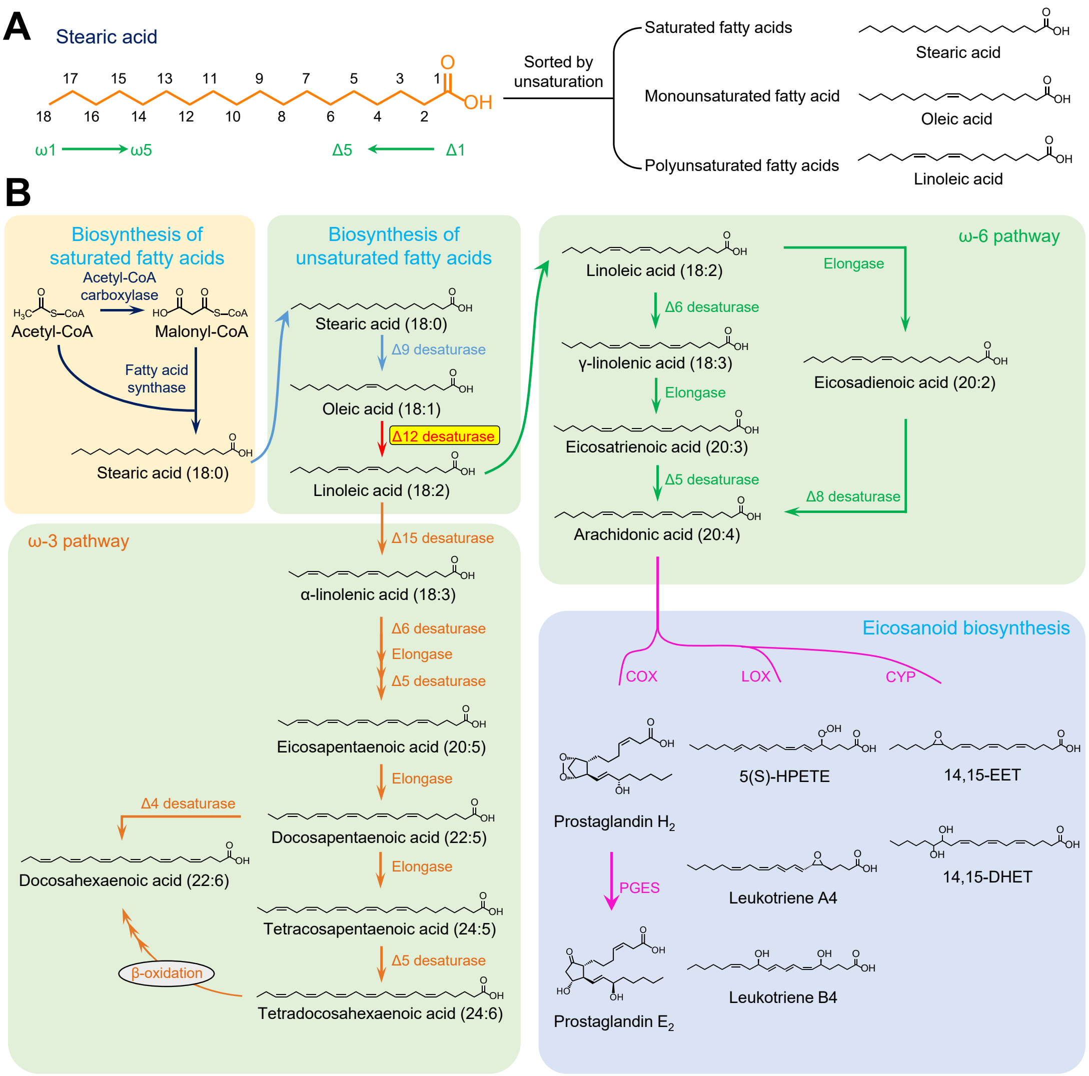

Figure S1. Representative fatty acid classification and biosynthesis. A) A diagram using stearic acid (18:0) as an example shows two fatty acid synthesis pathways of fatty acids from the methyl end (ω) and the carboxyl end (Δ) respectively. Fatty acids are classified into three types according to their unsaturation, represented by stearic, oleic and linoleic acids. B) Simplified pathways of fatty acid biosynthesis, including eicosanoid metabolites in rectangles. Biosynthesis of saturated fatty acids in yellow, biosynthesis of unsaturated fatty acids in green, eicosanoid metabolites in blue, ω-3 fatty acid pathway catalytic steps by the orange arrow, and ω-6 fatty acid pathway catalytic steps by the green arrow.

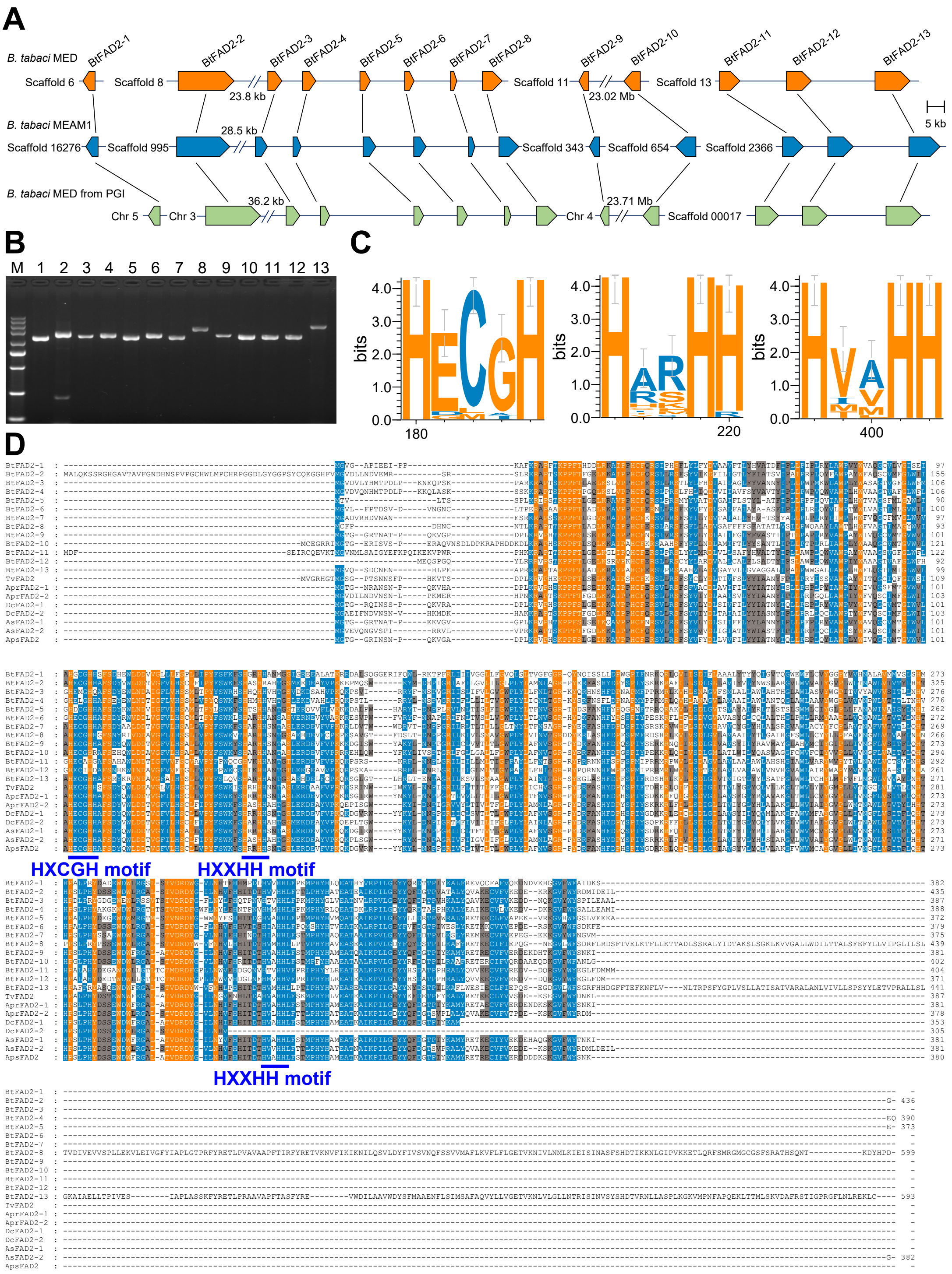

Figure S2. Genome-wide cloning and identification of *BtFAD2* gene family. A) Synteny analysis of 13 FAD2 genes among *B. tabaci* MED (Genome website: www.ncbi.nlm.nih.gov/nuccore/LIED00000000.1/), MEAM1 (Genome website: www.whiteflygenomics.org/cgi-bin/bta/index.cgi) and MED on chromosome level

(Genome website: www.pestgenomics.org/species/bemisia-tabaci). B) Full-length cDNA clones of all *BtFAD2* genes. M, marker (from top to bottom: 5,000 bp, 3,000 bp, 2,000 bp, 1,500 bp, 1,000 bp, 750 bp, 500 bp, 250 bp, 100 bp); lanes 1–13, PCR products of *BtFAD2-1* to *BtFAD2-13*. C) WebLogo plots highlight amino acid conservation in the three histidine clusters in all of the BtFAD2 and Aleyrodinae FAD2 proteins. D) Amino acid sequence alignment of above proteins. Conservative structural domains are marked with blue underlines.

A

Proteins	BtFAD2-1	BtFAD2-2	BtFAD2-3	BtFAD2-4	BtFAD2-5	BtFAD2-6	BtFAD2-7	BtFAD2-8	BtFAD2-9	BtFAD2-10	BtFAD2-11	BtFAD2-12	BtFAD2-13	TvFAD2	AprFAD2-1	AprFAD2-2	DcFAD2-1	DcFAD2-2	AsFAD2-1	AsFAD2-2	ApsFAD2	AtFAD2
BtFAD2-1	100%																					
BtFAD2-2	40%	100%																				
BtFAD2-3	41%	42%	100%																			
BtFAD2-4	40%	42%	70%	100%																		
BtFAD2-5	42%	44%	44%	44%	100%																	
BtFAD2-6	45%	46%	48%	45%	54%	100%																
BtFAD2-7	47%	53%	46%	45%	52%	58%	100%															
BtFAD2-8	26%	28%	27%	27%	31%	32%	34%	100%														
BtFAD2-9	47%	54%	45%	45%	53%	57%	62%	33%	100%													
BtFAD2-10	47%	49%	42%	43%	46%	52%	54%	32%	63%	100%												
BtFAD2-11	37%	43%	52%	53%	44%	43%	44%	24%	43%	43%	100%											
BtFAD2-12	39%	41%	53%	52%	45%	45%	44%	25%	44%	42%	71%	100%										
BtFAD2-13	25%	28%	27%	27%	31%	30%	34%	54%	31%	31%	25%	27%	100%									
TvFAD2	45%	50%	45%	44%	49%	54%	55%	29%	65%	53%	42%	42%	29%	100%								
AprFAD2-1	46%	53%	45%	45%	52%	59%	63%	33%	81%	62%	43%	43%	32%	65%	100%							
AprFAD2-2	46%	69%	50%	51%	51%	56%	63%	31%	61%	53%	45%	45%	32%	62%	63%	100%						
DcFAD2-1	44%	50%	43%	44%	49%	55%	59%	31%	78%	58%	41%	42%	30%	62%	79%	61%	100%					
DcFAD2-2	37%	54%	37%	40%	38%	45%	50%	25%	47%	42%	34%	34%	25%	50%	50%	70%	54%	100%				
AsFAD2-1	46%	52%	47%	47%	53%	58%	62%	33%	85%	62%	43%	44%	32%	66%	81%	62%	80%	49%	100%			
AsFAD2-2	46%	74%	49%	51%	49%	54%	60%	32%	61%	54%	47%	47%	31%	57%	61%	82%	59%	64%	61%	100%		
ApsFAD2	47%	54%	47%	46%	53%	59%	63%	32%	84%	62%	44%	45%	32%	67%	86%	66%	87%	51%	85%	63%	100%	
AtFAD2	45%	49%	46%	47%	49%	52%	59%	31%	69%	56%	44%	42%	30%	61%	70%	61%	67%	47%	71%	58%	72%	100%

Sequence similarity

<20%	>20%	>40%	>60%	>80%	100%

B

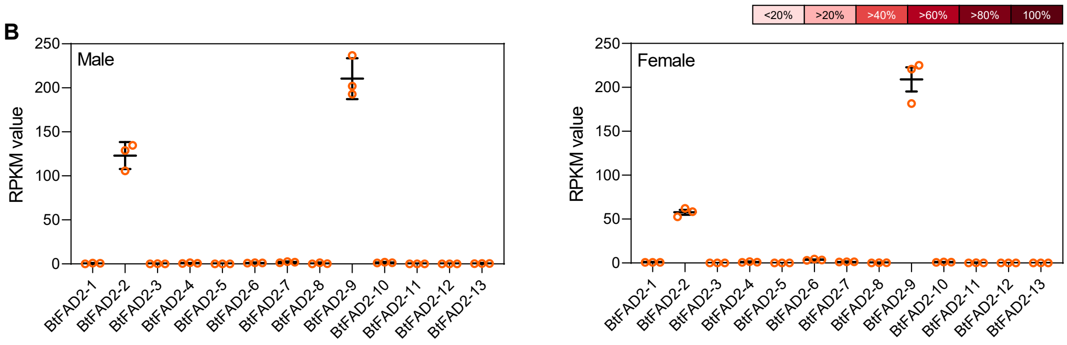

Figure S3. Similarity and RPKM value of *BtFAD2* gene family. A) Pairwise comparison of amino acid sequence identities among all the identified BtFAD2 proteins, Aleyrodinae FAD2 proteins and *Arabidopsis thaliana* FAD2 protein (NP_001319529.1). Values in each rectangle represent percent similarity. Percentage similarity for each comparison is color-coded according to the gradient at the bottom. B) The RPKM value of *BtFAD2* genes in male and female *B. tabaci* MED adults as determined by analyzing our previous *B. tabaci* MED transcriptome libraries.

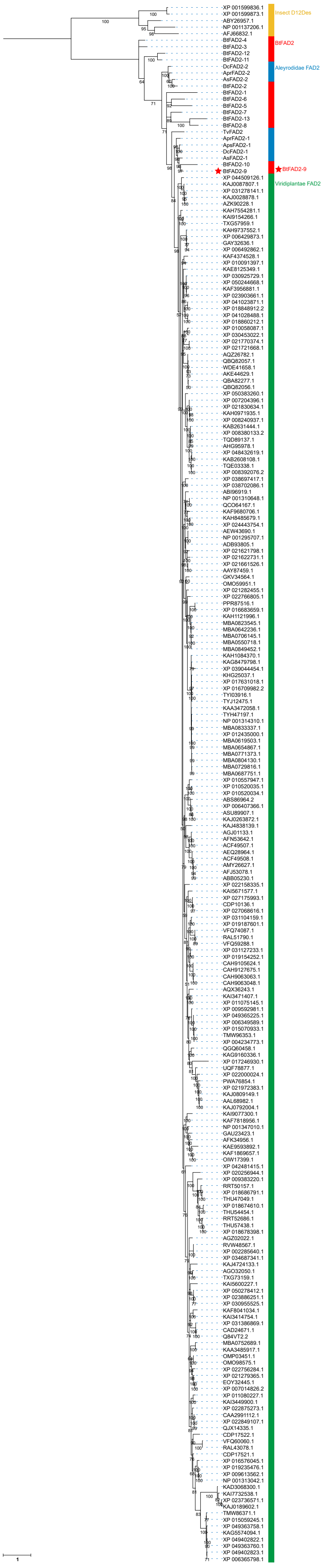
Insect D12Des
BtFAD2
Aleyrodidae FAD2
BtFAD2-9
Viridiplantae FAD2
1

Figure S4. Detailed view of the Bayesian-based phylogenetic analysis of BtFAD2, related to Figure 1C. A red, blue and green colors indicate *B. tabaci*, Aleyrodinae and plant FAD2s, respectively, while a yellow color indicates insect Δ12 desaturases (Figure 1C). Sequences corresponding to accession numbers or enzyme names in the tree can be extracted from the GenBank database (Table S2 and Table S3, Supporting Information). BtFAD2-9 is indicated by a red star. The tree was rooted by midpoint rooting method. The scale bar represents 1 amino acid substitutions per site.

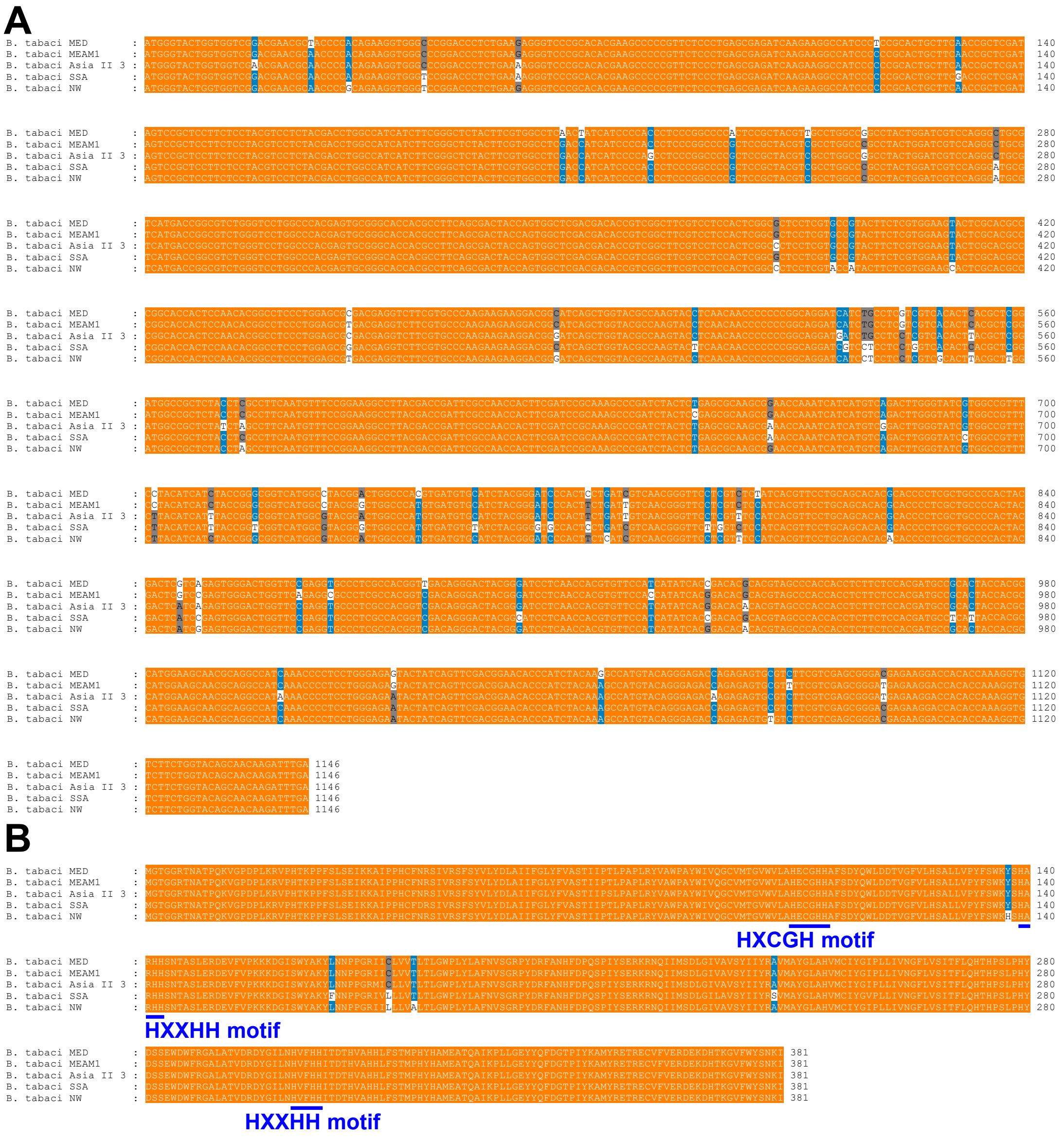

Figure S5. Nucleotide and amino acid sequence comparison of *BtFAD2-9* in different cryptic species of *B. tabaci* (MED, MEAM1, Asia II, SSA and NW). A) Nucleic acid sequence alignment of *BtFAD2-9*. B) Amino acid sequence alignment of *BtFAD2*. The species names are indicated on the left.

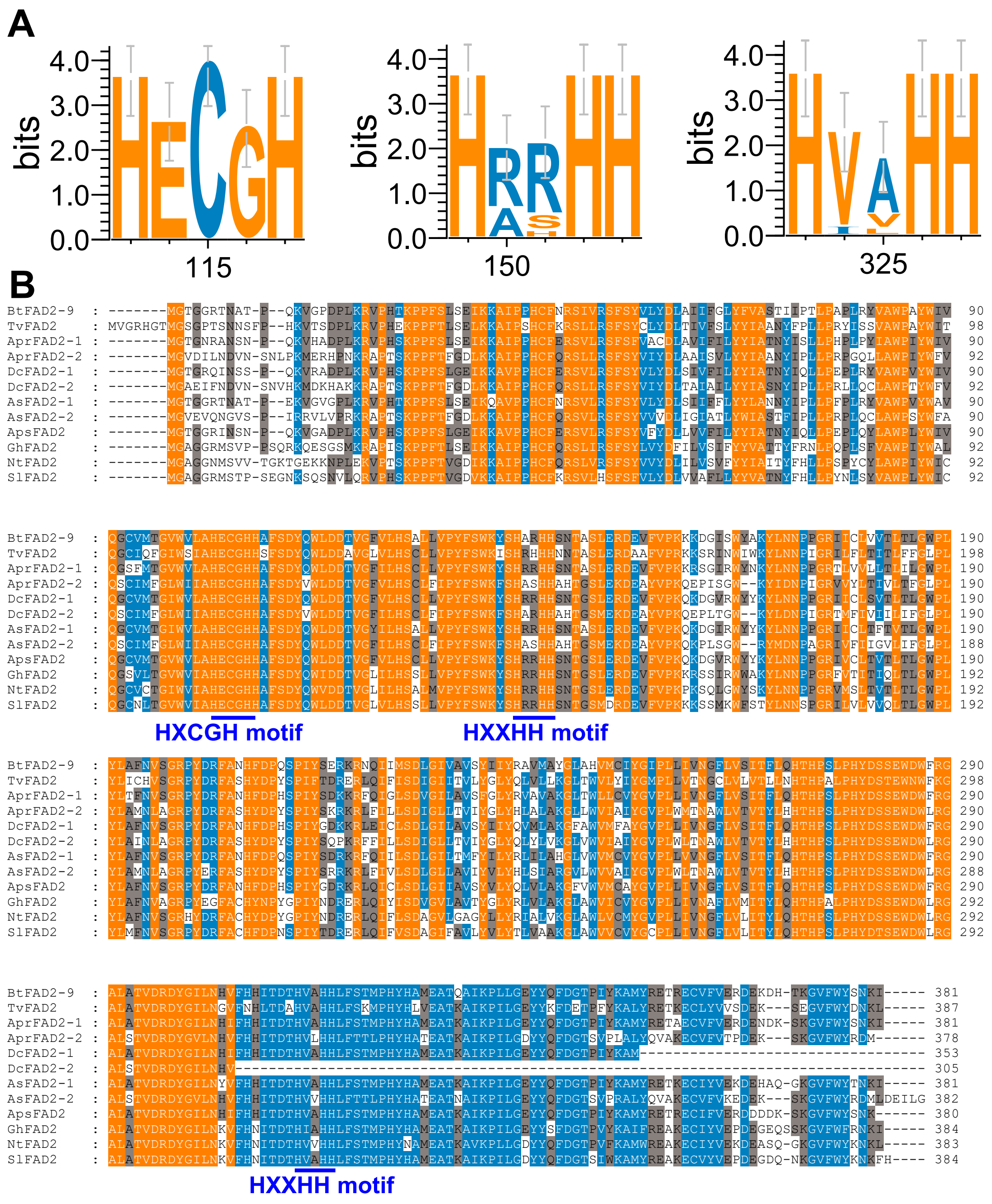

Figure S6. Amino acid sequence alignment of BtFAD2-9, Aleyrodinae FAD2 and its homologs in different host plant species. A) WebLogo plots highlight amino acid conservation in the three histidine clusters in BtFAD2-9, Aleyrodinae FAD2 and host plant homologs of BtFAD2-9 related to (B). B) Amino acid sequence alignment of above proteins. Conservative structural domains are marked with blue underlines. Gene names are indicated on the left, and the highly conserved HXCGH, HXXHH

and HXXHH motifs are denoted. Host plant homologs of BtFAD2-9 were retrieved from the GenBank. GhFAD2 (*Gossypium hirsutum*, NP_001314310.1); NtFAD2 (*Nicotiana tabacum*, NP_001313042.1); SlFAD2 (*Solanum lycopersicum*, XP_004234773.1).

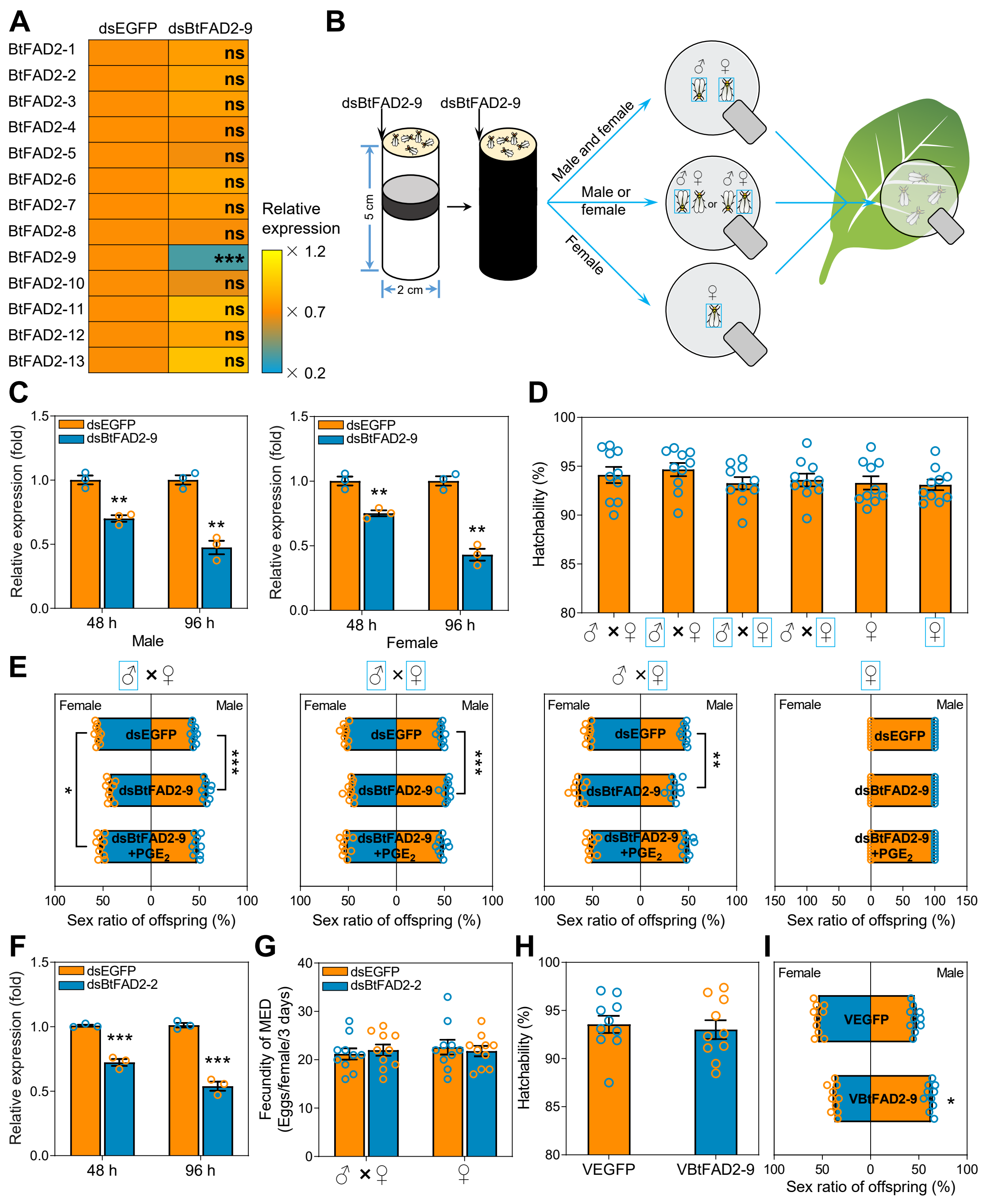

Figure S7. RNAi and VIGS assays of *BtFAD2* in *B. tabaci*. A) The transcript level of 13 *BtFAD2* genes at 96 h feeding dsBtFAD2-9 as determined by qPCR. B) Schematic representation of the experiment design. A diagram of the whitefly dsRNA feeding setup was built. Then, *B. tabaci* adults were set in four treatments in clip cage: dsBtFAD2-9 ♂ ×dsBtFAD2-9 ♀; dsBtFAD2-9 ♂ × control ♀; control ♂ ×dsBtFAD2-9 ♀; dsBtFAD2-9 ♀ for parthenogenesis. Blue boxes indicate

RNAi-treated whiteflies. Finally, the clip cage was put on the tobacco leaves. C) The transcript levels of *BtFAD2-9* in male and female whitefly at 48 h and 96 h post-RNAi as determined by qPCR, respectively. D) The hatching rate of whitefly progeny produced from the above mating treatments in (B). E) The sex ratio of whitefly progeny produced from the above mating treatments in (B). F) The transcript levels of *BtFAD2-2* at 48 h and 96 h post-RNAi as determined by qPCR. G) Sexual and parthenogenetic fecundity of *B. tabaci* feeding with dsBtFAD2-2 for 96 h. H) The hatchability of whitefly progeny feeding on VIGS plants. I) The sex ratio of whitefly progeny feeding on VIGS plants. Values are means ± SEM, $n = 3$ (A, C, F), n = 10 (D, E, G-I) biologically independent samples, ns, $P > 0.05$, *$P < 0.05$, **$P < 0.01$, ***$P < 0.001$, one-way ANOVA with Tukey's test was used for comparison.

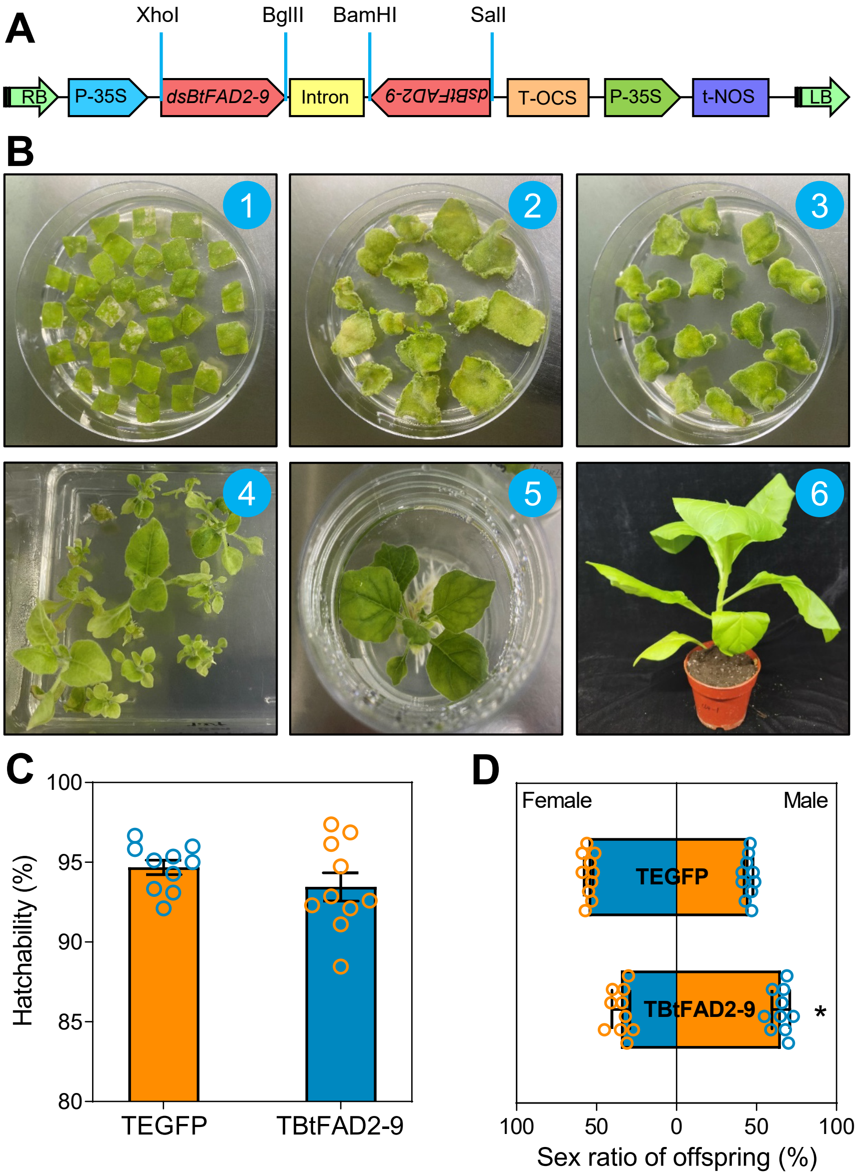

Figure S8. The dsRNA-expressing vector, genetic transformation, and regeneration of transgenic tobacco lines. A) The schematic representation of the pCAMBIA-RNAi-*BtFAD2-9* expression cassette used for tobacco transformation. B) Schematic representation of constructing transgenic tobacco. C) The hatching rate of whitefly progeny feeding on transgenic plants. D) The sex ratio of whitefly progeny feeding on transgenic plants. Values are means ± SEM, n = 10 (C, D) biologically independent samples, *$P < 0.05$, **$P < 0.01$, ***$P < 0.001$, one-way ANOVA with Tukey's test was used for comparison.

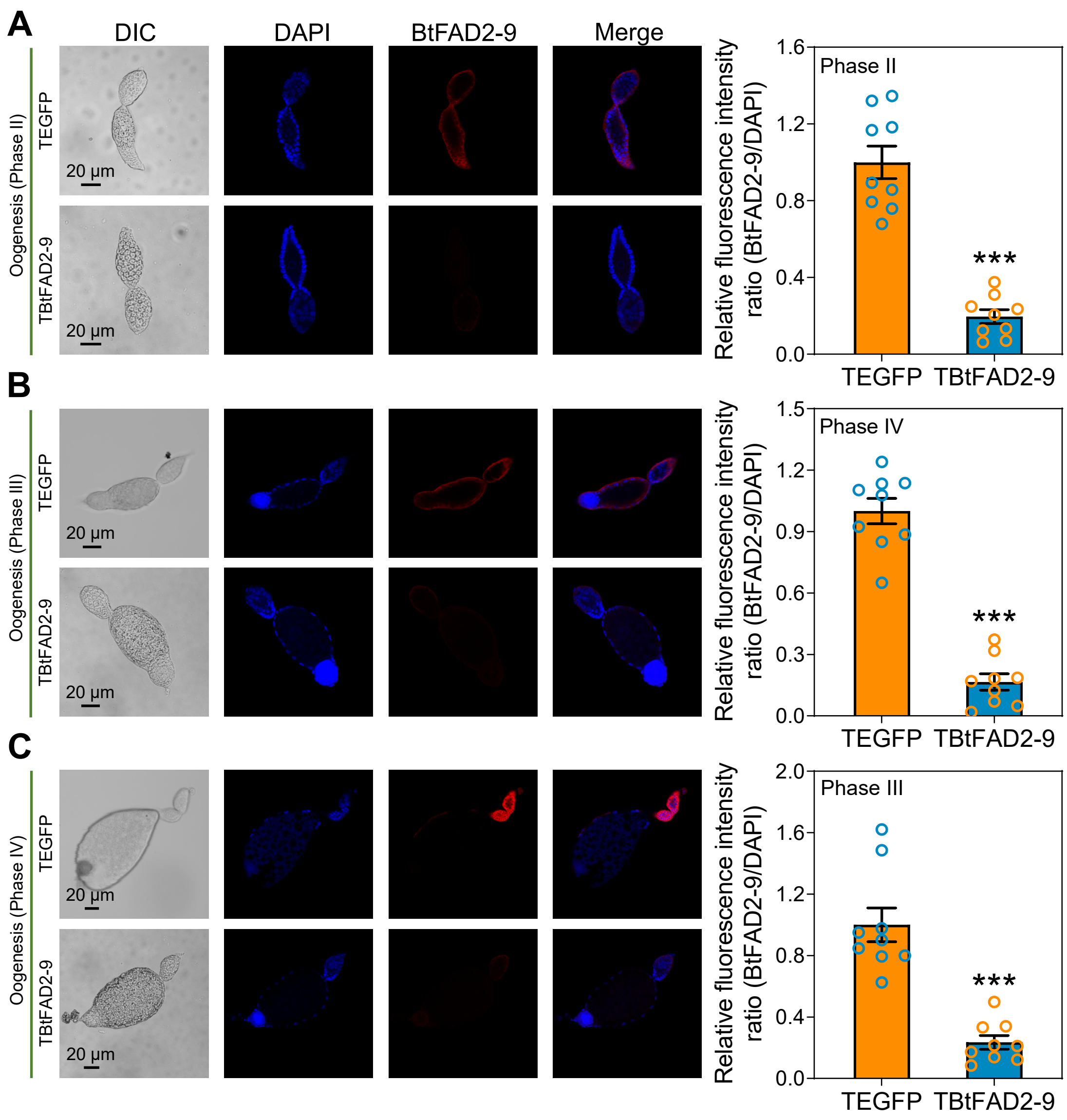

Figure S9. Localization of BtFAD2-9 protein in oogenesis of female whitefly after feeding on transgenic plants. A-C) Localization of BtFAD2-9 protein in oogenesis phase II (A), III (B) and IV (C) of female whitefly after feeding on transgenic-BtFAD2-9 tobacco and transgenic-EGFP tobacco for 7 days. Nuclei are stained with DAPI (blue), red is the positive signal for anti-BtFAD2-9. Relative fluorescence intensity ratio was quantified by ImageJ v.1.51. Values are means ± SEM, n = 9 (A-C) biologically independent samples, *$P < 0.05$, **$P < 0.01$, ***$P < 0.001$, one-way ANOVA with Tukey's test was used for comparison.

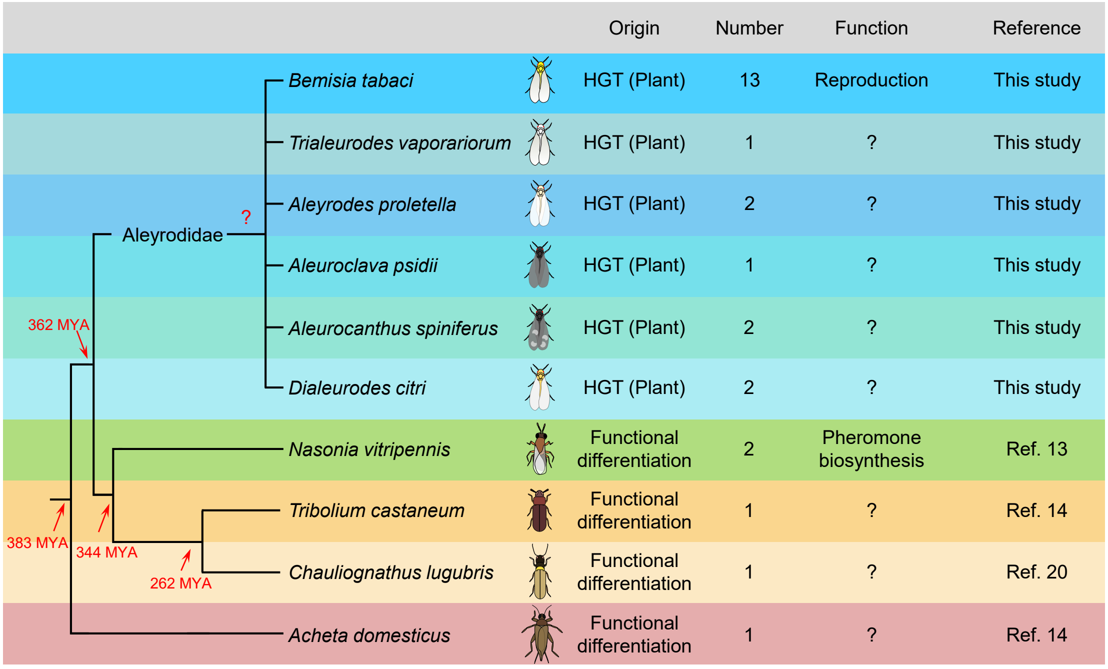

Figure S10. Comparison of genes functioning as Δ12 fatty acid desaturase in different insects. Million years ago (MYA) values indicate when insects diverged (based on the TimeTree, http://www.timetree.org/). All information in this overview was derived from literature or this study.

Table S1. Primers used in this study

Application	Gene	Primers	Primer sequence (5′-3′)	Tm (°C)	PCR product (bp)
Gene cloning	BtFAD2-1	cBtFAD2-1-F	ACAAGTATGGGCGTAGGAG	55	1167
		cBtFAD2-1-R	CATGCCAGAAGATCATGATT		
	BtFAD2-2	cBtFAD2-2-F	ATGGCGCTCCAGAAGTCC	55	1163
		cBtFAD2-2-R	AGCCTCCATCTTACCCGAG		
	BtFAD2-3	cBtFAD2-3-F	GAGGTCAGAAGTATGGGTGTG	55	1188
		cBtFAD2-3-R	GGAGCTTTCAACTCAGAGTGC		
	BtFAD2-4	cBtFAD2-4-F	TAGATTAGAATCATGGGTGTGG	55	1215
		cBtFAD2-4-R	TTACATAGAAATTAGGATCGAAGAA		
	BtFAD2-5	cBtFAD2-5-F	TTTCAAAATGACAGTATTGACCTC	55	1259
		cBtFAD2-5-R	AATAAAATTCCGACTTAGCGC		
	BtFAD2-6	cBtFAD2-6-F	GAATGGGCGTGCTTTTCC	58	1171
		cBtFAD2-6-R	CTACCTATCTGACCAACTCTAGCAA		
	BtFAD2-7	cBtFAD2-7-F	GAGGACCAGGATATGGGAGC	58	1153
		cBtFAD2-7-R	CCCCGCCCTCAAATTACA		
	BtFAD2-8	cBtFAD2-8-F	ACATTCAAGATGTGTTTCCC	52	1518
		cBtFAD2-8-R	TTTCAATCCGGGTGGTAG		
	BtFAD2-9	cBtFAD2-9-F	CTGAAAAAATGGGTACTGGT	52	1163
		cBtFAD2-9-R	GAATCATCATCAAATCTTGTTG		
	BtFAD2-10	cBtFAD2-10-F	GCGTGGAGTATGTGCGAA	55	1227
		cBtFAD2-10-R	GCTGTGATCTCAACCCAGATT		
	BtFAD2-11	cBtFAD2-11-F	ATGGATTTTTCTGAAATTCGG	56	1215
		cBtFAD2-11-R	TCACATCATCATGTCGAAGAGTC		
	BtFAD2-12	cBtFAD2-12-F	ATGGAGCAGAGCCCTGGAC	59	1116
		cBtFAD2-12-R	CTACATGTCGAAGAGTCCCTCG		
	BtFAD2-13	cBtFAD2-13-F	ATGGGCGTCCAGTCAGACT	55	1782
		cBtFAD2-13-R	CTAGCATAGTTTCTCTCTTAGATTG		
qPCR	BtFAD2-1	qBtFAD2-1-F	ATCTTGGGCGAGTATTATCAGC	60	129

		qBtFAD2-1-R	GATGGCAAACCAGAATACACCT		
	BtFAD2-2	qBtFAD2-2-F	TTCATCGCCACAAGTGTCATT	58	107
		qBtFAD2-2-R	ATGATCCACAGGCCAAACA		
	BtFAD2-3	qBtFAD2-3-F	CAGGCATTTAGCGACTACGAA	58	142
		qBtFAD2-3-R	GAGCAGAATCTTTATCAACGGA		
	BtFAD2-4	qBtFAD2-4-F	CAACAGCTTCCTCGATCCG	58	130
		qBtFAD2-4-R	AGCGAGTCCCTGCGTGTTA		
	BtFAD2-5	qBtFAD2-5-F	CGTTCAGTGACTACAAGTGGCT	58	150
		qBtFAD2-5-R	TTTTCACCAAGAATACGACCTGT		
	BtFAD2-6	qBtFAD2-6-F	CGTACTTTTCGTGGAAGCTCT	56	128
		qBtFAD2-6-R	GGCGTTGTTGAAGTACCTGA		
	BtFAD2-7	qBtFAD2-7-F	TTCAAACGTTCCGTCCTGC	59	96
		qBtFAD2-7-R	TAGCAGTGCGTAATACGAAGTGA		
	BtFAD2-8	qBtFAD2-8-F	ATCACTTGTTGCCTACCGTACC	58	92
		qBtFAD2-8-R	GGAGTCATATTGGAAATAGTCACCT		
	BtFAD2-9	qBtFAD2-9-F	CACCTCTTCTCCACGATGC	56	105
		qBtFAD2-9-R	CTTGTAGATGGGTGTTCCGTC		
	BtFAD2-10	qBtFAD2-10-F	GGAGGGTTCTGCTGGGTCTT	56	125
		qBtFAD2-10-R	CACTCGGAGGAGTCGTAGTGC		
	BtFAD2-11	qBtFAD2-11-F	ATTCGGTGTCAGGAGGTCAAA	59	147
		qBtFAD2-11-R	TTCTCCAAGGGTGAAGGGG		
	BtFAD2-12	qBtFAD2-12-F	CTCTTCCTCTCGGCTGACAC	58	144
		qBtFAD2-12-R	GCTGAAGGCGTCGTGGAT		
	BtFAD2-13	qBtFAD2-13-F	CCCATCACATCACGGACAC	56	125
		qBtFAD2-13-R	CGTCGAGTCGTAGTTGTAGTAGG		
	BtEF1-α	qBtEF1-α-F	TAGCCTTGTGCCAATTTCCG	60	110
		qBtEF1-α-R	CCTTCAGCATTACCGTCC		
Genome fragment	BtFAD2-9-upstream	upBtFAD2-9-1-F	TTTTGTCAACTATTGCTGGAAGTT	58	4438
		upBtFAD2-9-1-R	CGTTTTAATGGAATCCGTTTAGA		

cloning		upBtFAD2-9-2-F	ATACGAGGAGCTCTAAACGGATT	58	4466
		upBtFAD2-9-2-R	ATAGCGGTAGCAGTACCGGAG		
		upBtFAD2-9-3-F	AGTGAGTGAGTTTACAGAATGCTCC	59	4467
		upBtFAD2-9-3-R	CTTCAAACAGAAGATAGGCAACA		
		upBtFAD2-9-4-F	TAATCGCGACTGTATCGAGTGTT	59	4470
		upBtFAD2-9-4-R	CATTATTTCCACAGGCCAAGT		
		upBtFAD2-9-5-F	ACAACACTCCGATTCAAAAGCA	60	4477
		upBtFAD2-9-5-R	AGTCCTAGTGACTGGATGTCGGT		
	BtFAD2-9-downstream	downBtFAD2-9-1-F	CCTCATCACTAAAGGATAGAAAGTA	56	2376
		downBtFAD2-9-1-R	TCTGGGAGACATCCGCTTA		
Transgenic yeast[a)]	BtFAD2-9	pyBtFAD2-9-F	**CTATAGGGAATATTAACCATGG**CTGAAAAAATGGGTACTGGT	52	1164
		pyBtFAD2-9-R	**GATATCTGCAGAATT**GAATCATCATCAAATCTTGTTG		
RNAi[b)]	dsBtFAD2-2	dsBtFAD2-2-F	**TAATACGACTCACTATAGGGAGA**GGCTCCTCGCTGTCATCTAC		
		dsBtFAD2-2-R	**TAATACGACTCACTATAGGGAGA**TCACGAAAACGCACTCTTTG		
	dsBtFAD2-9	dsBtFAD2-9-F	**TAATACGACTCACTATAGGGAGA**CTTCGTCCTCCACTCGGC	60	537
		dsBtFAD2-9-R	**TAATACGACTCACTATAGGGAGA**CCGTAGTCCCTGTCAACCGT		
	dsEGFP	dsEGFP-F	**TAATACGACTCACTATAGGGAGA**CCACAAGTTCAGCGTGTCCG	55	435
		dsEGFP-R	**TAATACGACTCACTATAGGGAGA**AAGTTCACCTTGATGCCGTTC		
VIGS[c)]	dsBtFAD2-9	vBtFAD2-9-F	**GCCTCGAGACGCGTG**CTTCGTCCTCCACTCGGC	60	537
		vBtFAD2-9-R	**GAGGCCTTCTAGAGA**CCGTAGTCCCTGTCAACCGT		
	dsEGFP	vEGFP-F	**GCCTCGAGACGCGTG**CCACAAGTTCAGCGTGTCCG	55	435
		vEGFP-R	**GAGGCCTTCTAGAGA**AAGTTCACCTTGATGCCGTTC		

Transgenic fly	β-tubulin	Tubulin-F	CTAAACCACGTAGCCGAGCC	60	455
		Tubulin-R	GTCGGGGTACTCCTCGCGGA		
Transgenic plants[d)]	dsBtFAD2-9	Sense-BtFAD2-9-F	**TGAGGATCCGAATTC**CTTCGTCCTCCACTCGGC	60	537
		Sense-BtFAD2-9-R	**GATCATTCAAAAGAC**CCGTAGTCCCTGTCAACCGT		
		Anti-sense-BtFAD2-9-F	**CCGGGTACCGAGCTC**CTTCGTCCTCCACTCGGC	60	537
		Anti-sense-BtFAD2-9-R	**TTGATTTTCTGCGCA**CCGTAGTCCCTGTCAACCGT		
	dsEGFP	Sense-EGFP-F	**TGAGGATCCGAATTC**CCACAAGTTCAGCGTGTCCG	55	435
		Sense-EGFP-R	**GATCATTCAAAAGAC**AAGTTCACCTTGATGCCGTTC		
		Anti-sense-EGFP-F	**CCGGGTACCGAGCTC**CCACAAGTTCAGCGTGTCCG	55	435
		Anti-sense-EGFP-R	**TTGATTTTCTGCGCA**AAGTTCACCTTGATGCCGTTC		

[a), b), c)]The adaptors are underlined. Their lengths were not calculated in the length of final PCR product.

[d)]The T7 promoter sequence is underlined. Their lengths were not calculated in the length of final PCR product.

Table S2. List of the genes used in this study

Name[a)]	Species	Annotation[b)]	Source	Gene ID[c)]	Size (aa)	Sequence quality	Used in tree[d)]
Plant							
AanFAD2	*Artemisia annua*	delta(12)-fatty-acid desaturase	GenBank	PWA76854.1	383	Complete	Yes
AarFAD2	*Ambrosia artemisiifolia*	uncharacterized protein	GenBank	KAI7732538.1	377	Complete	Yes
AnFAD2	*Acer negundo*	uncharacterized protein	GenBank	KAI9154266.1	465	Complete	Yes
AoFAD2	*Asparagus officinalis*	delta(12)-fatty-acid desaturase	GenBank	XP_020256944.1	383	Complete	Yes
ApFAD2	*Acacia pycnantha*	uncharacterized protein	GenBank	KAI9077300.1	383	Complete	Yes
AyFAD2-1	*Acer yangbiense*	uncharacterized protein	GenBank	TXG73159.1	386	Complete	Yes
AyFAD2-2	*Acer yangbiense*	uncharacterized protein	GenBank	TXG57959.1	383	Complete	Yes
BaFAD2	*Buglossoides arvensis*	delta(12)-fatty-acid desaturase	GenBank	QGQ60458.1	383	Complete	Yes
CanFAD2	*Capsicum annuum*	delta(12)-fatty-acid desaturase	GenBank	XP_016576045.1	384	Complete	Yes
CarFAD2	*Coffea arabica*	delta(12)-fatty-acid desaturase	GenBank	XP_027068616.1	383	Complete	Yes
CauFAD2-1	*Cuscuta australis*	uncharacterized protein	GenBank	RAL51790.1	375	Complete	Yes
CauFAD2-2	*Cuscuta australis*	uncharacterized protein	GenBank	RAL43078.1	383	Complete	Yes
CcFAD2	*Citrus clementina*	delta(12)-fatty-acid desaturase	GenBank	XP_006429873.1	383	Complete	Yes
CciFAD2	*Corymbia citriodora*	uncharacterized protein	GenBank	KAF8041034.1	379	Complete	Yes
CcmFAD2-1	*Cuscuta campestris*	uncharacterized protein	GenBank	VFQ59288.1	375	Complete	Yes
CcmFAD2-2	*Cuscuta campestris*	uncharacterized protein	GenBank	VFQ74087.1	369	Complete	Yes
CcmFAD2-3	*Cuscuta campestris*	uncharacterized protein	GenBank	VFQ60060.1	383	Complete	Yes
CcnFAD2-1	*Coffea canephora*	uncharacterized protein	GenBank	CDP10136.1	458	Complete	Yes
CcnFAD2-2	*Coffea canephora*	uncharacterized protein	GenBank	CDP17521.1	381	Complete	Yes
CcnFAD2-3	*Coffea canephora*	uncharacterized protein	GenBank	CDP17522.1	381	Complete	Yes
CcpFAD2	*Corchorus capsularis*	delta(12)-fatty-acid desaturase	GenBank	OMO98575.1	383	Complete	Yes

CegFAD2	*Coffea eugenioides*	delta(12)-fatty-acid desaturase	GenBank	XP_027175993.1	383	Complete	Yes
CepFAD2-1	*Cuscuta epithymum*	uncharacterized protein	GenBank	CAH9127675.1	385	Complete	Yes
CepFAD2-2	*Cuscuta epithymum*	uncharacterized protein	GenBank	CAH9105624.1	385	Complete	Yes
CerFAD2	*Cuscuta europaea*	uncharacterized protein	GenBank	CAH9063048.1	385	Complete	Yes
CerFAD2	*Cuscuta europaea*	uncharacterized protein	GenBank	CAH9063063.1	385	Complete	Yes
CfFAD2	*Carpinus fangiana*	uncharacterized protein	GenBank	KAE8125349.1	383	Complete	Yes
CmFAD2	*Castanea mollissima*	uncharacterized protein	GenBank	KAF3956881.1	383	Complete	Yes
CoFAD2-1	*Corchorus olitorius*	delta(12)-fatty-acid desaturase	GenBank	OMO59951.1	384	Complete	Yes
CoFAD2-2	*Corchorus olitorius*	delta(12)-fatty-acid desaturase	GenBank	OMP03451.1	383	Complete	Yes
CqFAD2-1	*Chenopodium quinoa*	delta(12)-fatty-acid desaturase	GenBank	XP_021721668.1	382	Complete	Yes
CqFAD2-2	*Chenopodium quinoa*	delta(12)-fatty-acid desaturase	GenBank	XP_021770374.1	382	Complete	Yes
CrFAD2	*Catharanthus roseus*	uncharacterized protein	GenBank	KAI5671577.1	384	Complete	Yes
CsFAD2	*Cannabis sativa*	uncharacterized protein	GenBank	KAF4374528.1	383	Complete	Yes
CsFAD2-1	*Citrus sinensis*	delta(12)-fatty-acid desaturase	GenBank	XP_006492862.1	383	Complete	Yes
CsFAD2-2	*Citrus sinensis*	delta(12)-fatty-acid desaturase	GenBank	KAH9737552.1	383	Complete	Yes
CuFAD2	*Citrus unshiu*	uncharacterized protein	GenBank	GAY32636.1	455	Complete	Yes
DcaFAD2	*Daucus carota*	delta(12)-fatty-acid desaturase	GenBank	XP_017246930.1	383	Complete	Yes
DsFAD2	*Descurainia sophia*	delta(12)-fatty-acid desaturase	GenBank	ABS86964.2	383	Complete	Yes
DzFAD2-1	*Durio zibethinus*	delta(12)-fatty-acid desaturase	GenBank	XP_022756284.1	383	Complete	Yes
DzFAD2-2	*Durio zibethinus*	delta(12)-fatty-acid desaturase	GenBank	XP_022766805.1	383	Complete	Yes
EgFAD2	*Eucalyptus grandis*	delta(12)-fatty-acid desaturase	GenBank	XP_010058087.1	383	Complete	Yes
EsFAD2	*Eutrema salsugineum*	delta(12)-fatty-acid desaturase	GenBank	XP_006407366.1	383	Complete	Yes
EvFAD2-1	*Ensete ventricosum*	uncharacterized protein	GenBank	RRT52686.1	386	Complete	Yes
EvFAD2-2	*Ensete ventricosum*	uncharacterized protein	GenBank	RRT50157.1	380	Complete	Yes
GabFAD2-1	*Gossypium arboreum*	delta(12)-fatty-acid desaturase	GenBank	XP_017631018.1	384	Complete	Yes
GabFAD2-2	*Gossypium arboreum*	delta(12)-fatty-acid desaturase	GenBank	KHG25037.1	392	Complete	Yes

GaiFAD2	*Gossypium aridum*	uncharacterized protein	GenBank	MBA0687751.1	384	Complete	Yes
GamFAD2-1	*Gossypium armourianum*	uncharacterized protein	GenBank	MBA0833337.1	384	Complete	Yes
GamFAD2-2	*Gossypium armourianum*	uncharacterized protein	GenBank	MBA0823545.1	383	Complete	Yes
GanFAD2	*Gossypium anomalum*	uncharacterized protein	GenBank	KAG8479798.1	384	Complete	Yes
GauFAD2-1	*Gossypium australe*	delta(12)-fatty-acid desaturase	GenBank	KAA3485917.1	383	Complete	Yes
GauFAD2-2	*Gossypium australe*	delta(12)-fatty-acid desaturase	GenBank	KAA3472058.1	384	Complete	Yes
GbFAD2	*Gossypium barbadense*	uncharacterized protein	GenBank	PPR87516.1	383	Complete	Yes
GdFAD2	*Gossypium davidsonii*	uncharacterized protein	GenBank	MBA0619503.1	384	Complete	Yes
GgFAD2	*Gossypium gossypioides*	uncharacterized protein	GenBank	MBA0752689.1	383	Complete	Yes
GhaFAD2	*Gossypium harknessii*	uncharacterized protein	GenBank	MBA0804130.1	384	Complete	Yes
GhFAD2-1	*Gossypium hirsutum*	delta(12)-fatty-acid desaturase	GenBank	NP_001314310.1	384	Complete	Yes
GhFAD2-2	*Gossypium hirsutum*	delta(12)-fatty-acid desaturase	GenBank	XP_016709982.2	384	Complete	Yes
GhFAD2-3	*Gossypium hirsutum*	delta(12)-fatty-acid desaturase	GenBank	XP_016683659.1	383	Complete	Yes
GkFAD2-1	*Gossypium klotzschianum*	uncharacterized protein	GenBank	MBA0654867.1	384	Complete	Yes
GkFAD2-2	*Gossypium klotzschianum*	uncharacterized protein	GenBank	MBA0642236.1	383	Complete	Yes
GlFAD2	*Gossypium lobatum*	uncharacterized protein	GenBank	MBA0550718.1	383	Complete	Yes
GlFAD2-1	*Gossypium laxum*	uncharacterized protein	GenBank	MBA0729816.1	384	Complete	Yes
GlFAD2-2	*Gossypium laxum*	uncharacterized protein	GenBank	MBA0706145.1	383	Complete	Yes
GmaFAD2	*Glycine max*	delta(12)-fatty-acid desaturase	GenBank	NP_001347010.1	383	Complete	Yes
GmuFAD2	*Gossypium mustelinum*	uncharacterized protein	GenBank	TYJ12475.1	384	Complete	Yes
GrFAD2	*Gossypium raimondii*	delta(12)-fatty-acid desaturase	GenBank	XP_012435000.1	384	Complete	Yes

GscFAD2	*Gossypium schwendimanii*	uncharacterized protein	GenBank	MBA0849452.1	383	Complete	Yes
GstFAD2-1	*Gossypium stocksii*	uncharacterized protein	GenBank	KAH1084370.1	450	Complete	Yes
GstFAD2-2	*Gossypium stocksii*	uncharacterized protein	GenBank	KAH1121996.1	383	Complete	Yes
GtoFAD2-1	*Gossypium tomentosum*	uncharacterized protein	GenBank	TYI03916.1	384	Complete	Yes
GtoFAD2-2	*Gossypium tomentosum*	uncharacterized protein	GenBank	TYH47197.1	384	Complete	Yes
GtrFAD2	*Gossypium trilobum*	uncharacterized protein	GenBank	MBA0771373.1	384	Complete	Yes
HaFAD2-1	*Helianthus annuus*	delta(12)-fatty-acid desaturase	GenBank	KAJ0809149.1	383	Complete	Yes
HaFAD2-2	*Helianthus annuus*	delta(12)-fatty-acid desaturase	GenBank	XP_022000024.1	382	Complete	Yes
HaFAD2-3	*Helianthus annuus*	delta(12)-fatty-acid desaturase	GenBank	KAJ0792004.1	383	Complete	Yes
HaFAD2-4	*Helianthus annuus*	delta(12)-fatty-acid desaturase	GenBank	XP_021972383.1	383	Complete	Yes
HaFAD2-5	*Helianthus annuus*	delta(12)-fatty-acid desaturase	GenBank	AAL68982.1	383	Complete	Yes
HbFAD2-1	*Hevea brasiliensis*	delta(12)-fatty-acid desaturase	GenBank	AAY87459.1	383	Complete	Yes
HbFAD2-2	*Hevea brasiliensis*	delta(12)-fatty-acid desaturase	GenBank	XP_021661526.1	383	Complete	Yes
HiFAD2	*Hirschfeldia incana*	delta(12)-fatty-acid desaturase	GenBank	KAJ0263872.1	383	Complete	Yes
HsFAD2	*Hibiscus syriacus*	delta(12)-fatty-acid desaturase	GenBank	XP_039044454.1	384	Complete	Yes
HuFAD2-1	*Herrania umbratica*	delta(12)-fatty-acid desaturase	GenBank	XP_021282455.1	383	Complete	Yes
HuFAD2-2	*Herrania umbratica*	delta(12)-fatty-acid desaturase	GenBank	XP_021279365.1	383	Complete	Yes
InFAD2-1	*Ipomoea nil*	delta(12)-fatty-acid desaturase	GenBank	XP_019187601.1	382	Complete	Yes
InFAD2-2	*Ipomoea nil*	delta(12)-fatty-acid desaturase	GenBank	XP_019154252.1	383	Complete	Yes
ItFAD2-1	*Ipomoea triloba*	delta(12)-fatty-acid desaturase	GenBank	XP_031127233.1	383	Complete	Yes
ItFAD2-2	*Ipomoea triloba*	delta(12)-fatty-acid desaturase	GenBank	XP_031104159.1	382	Complete	Yes
JcFAD2-1	*Jatropha curcas*	delta(12)-fatty-acid desaturase	GenBank	ADB93805.1	383	Complete	Yes
JcFAD2-2	*Jatropha curcas*	delta(12)-fatty-acid desaturase	GenBank	NP_001295707.1	383	Complete	Yes

JcFAD2-3	*Jatropha curcas*	delta(12)-fatty-acid desaturase	GenBank	AEW43690.1	383	Complete	Yes
JmFAD2-1	*Juglans microcarpa* x *Juglans regia*	delta(12)-fatty-acid desaturase	GenBank	XP_041028488.1	386	Complete	Yes
JmFAD2-2	*Juglans microcarpa* x *Juglans regia*	delta(12)-fatty-acid desaturase	GenBank	XP_041023871.1	383	Complete	Yes
JrFAD2-1	*Juglans regia*	delta(12)-fatty-acid desaturase	GenBank	XP_018860212.1	388	Complete	Yes
JrFAD2-2	*Juglans regia*	delta(12)-fatty-acid desaturase	GenBank	XP_018848912.2	383	Complete	Yes
KcFAD2	*Karelinia caspia*	delta(12)-fatty-acid desaturase	GenBank	UQF78877.1	383	Complete	Yes
LalFAD2-1	*Lupinus albus*	uncharacterized protein	GenBank	KAF1869657.1	401	Complete	Yes
LalFAD2-2	*Lupinus albus*	uncharacterized protein	GenBank	KAE9593892.1	413	Complete	Yes
LanFAD2	*Lupinus angustifolius*	uncharacterized protein	GenBank	OIW17399.1	386	Complete	Yes
LcFAD2	*Leucas cephalotes*	delta(12)-fatty-acid desaturase	GenBank	AQX36243.1	383	Complete	Yes
LeFAD2	*Lithospermum erythrorhizon*	uncharacterized protein	GenBank	KAG9160336.1	383	Complete	Yes
LgFAD2	*Linum grandiflorum*	delta(12)-fatty-acid desaturase	GenBank	AEQ28964.1	385	Complete	Yes
LjFAD2	*Lotus japonicus*	uncharacterized protein	GenBank	AFK34956.1	384	Complete	Yes
LsFAD2-1	*Lactuca sativa*	uncharacterized protein	GenBank	KAJ0189602.1	417	Complete	Yes
LsFAD2-2	*Lactuca sativa*	delta(12)-fatty-acid desaturase	GenBank	XP_023736571.1	377	Complete	Yes
LuFAD2-1	*Linum usitatissimum*	delta(12)-fatty-acid desaturase	GenBank	AGJ01133.1	382	Complete	Yes
LuFAD2-2	*Linum usitatissimum*	delta(12)-fatty-acid desaturase	GenBank	ACF49507.1	382	Complete	Yes
LuFAD2-3	*Linum usitatissimum*	delta(12)-fatty-acid desaturase	GenBank	AFN53642.1	508	Complete	Yes
LuFAD2-4	*Linum usitatissimum*	delta(12)-fatty-acid desaturase	GenBank	ACF49508.1	378	Complete	Yes
LuFAD2-5	*Linum usitatissimum*	delta(12)-fatty-acid desaturase	GenBank	ABB05230.1	378	Complete	Yes
LuFAD2-6	*Linum usitatissimum*	delta(12)-fatty-acid desaturase	GenBank	AMY26627.1	378	Complete	Yes
LuFAD2-7	*Linum usitatissimum*	delta(12)-fatty-acid desaturase	GenBank	AFJ53078.1	378	Complete	Yes
MaFAD2	*Melia azedarach*	delta(12)-fatty-acid desaturase	GenBank	KAJ4724133.1	383	Complete	Yes

MbcFAD2-1	*Malus baccata*	uncharacterized protein	GenBank	TQE03338.1	382	Complete	Yes
MbcFAD2-2	*Malus baccata*	delta(12)-fatty-acid desaturase	GenBank	AHG95978.1	382	Complete	Yes
MbcFAD2-3	*Malus baccata*	uncharacterized protein	GenBank	TQD89137.1	382	Complete	Yes
MblFAD2-1	*Musa balbisiana*	uncharacterized protein	GenBank	THU54454.1	370	Complete	Yes
MblFAD2-2	*Musa balbisiana*	uncharacterized protein	GenBank	THU47049.1	429	Complete	Yes
MblFAD2-3	*Musa balbisiana*	uncharacterized protein	GenBank	THU57438.1	386	Complete	Yes
McFAD2	*Momordica charantia*	delta(12)-fatty-acid desaturase	GenBank	XP_022158335.1	384	Complete	Yes
MdFAD2-1	*Malus domestica*	delta(12)-fatty-acid desaturase	GenBank	XP_008392076.2	382	Complete	Yes
MdFAD2-2	*Malus domestica*	delta(12)-fatty-acid desaturase	GenBank	XP_008380133.2	382	Complete	Yes
MdFAD2-3	*Mangifera indica*	delta(12)-fatty-acid desaturase	GenBank	XP_044509126.1	383	Complete	Yes
MeFAD2-1	*Manihot esculenta*	delta(12)-fatty-acid desaturase	GenBank	XP_021622731.1	381	Complete	Yes
MeFAD2-2	*Manihot esculenta*	delta(12)-fatty-acid desaturase	GenBank	XP_021621798.1	383	Complete	Yes
MiFAD2	*Macadamia integrifolia*	delta(12)-fatty-acid desaturase	GenBank	XP_042481415.1	388	Complete	Yes
MmFAD2	*Mikania micrantha*	uncharacterized protein	GenBank	KAD3068300.1	377	Complete	Yes
MmFAD2-1	*Musa acuminata* subsp. *malaccensis*	delta(12)-fatty-acid desaturase	GenBank	XP_018674610.1	395	Complete	Yes
MmFAD2-2	*Musa acuminata* subsp. *malaccensis*	delta(12)-fatty-acid desaturase	GenBank	XP_018678398.1	416	Complete	Yes
MmFAD2-3	*Musa acuminata* subsp. *malaccensis*	delta(12)-fatty-acid desaturase	GenBank	XP_009383220.1	375	Complete	Yes
MmFAD2-4	*Musa acuminata* subsp. *malaccensis*	delta(12)-fatty-acid desaturase	GenBank	XP_018686791.1	425	Complete	Yes
MnFAD2	*Morus notabilis*	delta(12)-fatty-acid desaturase	GenBank	XP_010091397.1	381	Complete	Yes
NaFAD2	*Nicotiana attenuata*	delta(12)-fatty-acid desaturase	GenBank	XP_019235476.1	383	Complete	Yes
NtFAD2	*Nicotiana tabacum*	delta(12)-fatty-acid desaturase	GenBank	NP_001313042.1	384	Complete	No

NtoFAD2-1	*Nicotiana tomentosiformis*	delta(12)-fatty-acid desaturase	GenBank	XP_009592981.1	384	Complete	Yes
NtoFAD2-2	*Nicotiana tomentosiformis*	delta(12)-fatty-acid desaturase	GenBank	XP_009613562.1	383	Complete	Yes
OeFAD2-1	*Olea europaea*	delta(12)-fatty-acid desaturase	GenBank	QJX14335.1	381	Complete	Yes
OeFAD2-2	*Olea europaea* subsp. *europaea*	delta(12)-fatty-acid desaturase	GenBank	CAA2991112.1	381	Complete	Yes
OeFAD2-3	*Olea europaea* var. *sylvestris*	delta(12)-fatty-acid desaturase	GenBank	XP_022849107.1	381	Complete	Yes
OeFAD2-4	*Olea europaea* var. *sylvestris*	delta(12)-fatty-acid desaturase	GenBank	XP_022875273.1	381	Complete	Yes
PanFAD2	*Potentilla anserina*	delta(12)-fatty-acid desaturase	GenBank	XP_050383260.1	382	Complete	Yes
ParFAD2	*Prunus armeniaca*	uncharacterized protein	GenBank	KAH0971935.1	382	Complete	Yes
PatFAD2	*Pistacia atlantica*	uncharacterized protein	GenBank	KAJ0087807.1	383	Complete	Yes
PavFAD2	*Prunus avium*	delta(12)-fatty-acid desaturase	GenBank	XP_021830634.1	382	Complete	Yes
PbFAD2	*Pyrus* x *bretschneideri*	delta(12)-fatty-acid desaturase	GenBank	XP_048432619.1	436	Complete	Yes
PcFAD2	*Pistacia chinensis*	delta(12)-fatty-acid desaturase	GenBank	AZK90228.1	383	Complete	Yes
PdaFAD2	*Paeonia delavayi*	delta(12)-fatty-acid desaturase	GenBank	AQZ26782.1	384	Complete	Yes
PdtFAD2	*Populus deltoides*	uncharacterized protein	GenBank	KAH8485679.1	385	Complete	Yes
PfFAD2-1	*Paulownia fortunei*	uncharacterized protein	GenBank	KAI3449900.1	383	Complete	Yes
PfFAD2-2	*Paulownia fortunei*	uncharacterized protein	GenBank	KAI3471407.1	383	Complete	Yes
PgFAD2-2	*Punica granatum*	delta(12)-fatty-acid desaturase	GenBank	Q84VT2.2	387	Complete	Yes
PgFAD2-3	*Punica granatum*	delta(12)-fatty-acid desaturase	GenBank	CAD24671.1	387	Complete	Yes
PgrFAD2-1	*Punica granatum*	delta(12)-fatty-acid desaturase	GenBank	XP_031386869.1	387	Complete	Yes
PguFAD2	*Psidium guajava*	uncharacterized protein	GenBank	KAI3414754.1	386	Complete	Yes
PiFAD2	*Pistacia integerrima*	uncharacterized protein	GenBank	KAJ0028878.1	467	Complete	Yes

PlaFAD2-1	*Paeonia lactiflora*	delta(12)-fatty-acid desaturase	GenBank	AKE44629.1	384	Complete	Yes
PlaFAD2-2	*Paeonia lactiflora*	delta(12)-fatty-acid desaturase	GenBank	WDE41658.1	384	Complete	Yes
PluFAD2	*Paeonia ludlowii*	delta(12)-fatty-acid desaturase	GenBank	QBQ82057.1	384	Complete	Yes
PmFAD2	*Prunus mume*	delta(12)-fatty-acid desaturase	GenBank	XP_008240937.1	382	Complete	Yes
PoFAD2	*Paeonia ostii*	delta(12)-fatty-acid desaturase	GenBank	QBQ82056.1	384	Complete	Yes
PpeFAD2	*Prunus persica*	delta(12)-fatty-acid desaturase	GenBank	XP_007204396.1	382	Complete	Yes
PpiFAD2	*Pongamia pinnata*	delta(12)-fatty-acid desaturase	GenBank	AGZ02022.1	385	Complete	Yes
PrFAD2	*Paeonia rockii*	delta(12)-fatty-acid desaturase	GenBank	QBA82277.1	384	Complete	Yes
PtFAD2-1	*Populus trichocarpa*	delta(12)-fatty-acid desaturase	GenBank	XP_024443754.1	385	Complete	Yes
PtFAD2-2	*Populus trichocarpa*	uncharacterized protein	GenBank	KAI5600227.1	382	Complete	Yes
PuFAD2-1	*Pyrus ussuriensis* x *Pyrus communis*	delta(12)-fatty-acid desaturase	GenBank	KAB2608108.1	382	Complete	Yes
PuFAD2-2	*Pyrus ussuriensis* x *Pyrus communis*	delta(12)-fatty-acid desaturase	GenBank	KAB2631444.1	382	Complete	Yes
PvFAD2	*Pistacia vera*	delta(12)-fatty-acid desaturase	GenBank	XP_031278141.1	383	Complete	Yes
QlFAD2-1	*Quercus lobata*	delta(12)-fatty-acid desaturase	GenBank	XP_030955525.1	382	Complete	Yes
QlFAD2-2	*Quercus lobata*	delta(12)-fatty-acid desaturase	GenBank	XP_030925729.1	383	Complete	Yes
QrFAD2-1	*Quercus robur*	delta(12)-fatty-acid desaturase	GenBank	XP_050244668.1	383	Complete	Yes
QrFAD2-2	*Quercus robur*	delta(12)-fatty-acid desaturase	GenBank	XP_050278412.1	382	Complete	Yes
QsFAD2-1	*Quercus suber*	delta(12)-fatty-acid desaturase	GenBank	XP_023903661.1	383	Complete	Yes
QsFAD2-2	*Quercus suber*	delta(12)-fatty-acid desaturase	GenBank	XP_023886251.1	382	Complete	Yes
RcFAD2-1	*Ricinus communis*	delta(12)-fatty-acid desaturase	GenBank	QCO64167.1	383	Complete	Yes
RcFAD2-2	*Ricinus communis*	delta(12)-fatty-acid desaturase	GenBank	NP_001310648.1	383	Complete	Yes
SaFAD2	*Sinapis alba*	delta(12)-fatty-acid desaturase	GenBank	ASU89907.1	383	Complete	Yes
SchFAD2-1	*Solanum chilense*	uncharacterized protein	GenBank	TMW96353.1	384	Complete	Yes
SchFAD2-2	*Solanum chilense*	uncharacterized protein	GenBank	TMW86371.1	379	Complete	Yes

ScoFAD2	*Solanum commersonii*	uncharacterized protein	GenBank	KAG5574094.1	395	Complete	Yes
SdFAD2	*Salix dunnii*	uncharacterized protein	GenBank	KAF9680706.1	384	Complete	Yes
SiFAD2-1	*Sesamum indicum*	delta(12)-fatty-acid desaturase	GenBank	XP_011075145.1	383	Complete	Yes
SiFAD2-2	*Sesamum indicum*	delta(12)-fatty-acid desaturase	GenBank	XP_011080227.1	383	Complete	Yes
SleFAD2	*Shorea leprosula*	uncharacterized protein	GenBank	GKV34564.1	382	Complete	Yes
SlFAD2	*Solanum lycopersicum*	delta(12)-fatty-acid desaturase	GenBank	XP_004234773.1	379	Complete	No
SoFAD2	*Syzygium oleosum*	delta(12)-fatty-acid desaturase	GenBank	XP_030453022.1	392	Complete	Yes
SpFAD2-1	*Solanum pennellii*	delta(12)-fatty-acid desaturase	GenBank	XP_015059245.1	379	Complete	Yes
SpFAD2-2	*Solanum pennellii*	delta(12)-fatty-acid desaturase	GenBank	XP_015070933.1	384	Complete	Yes
SsFAD2-1	*Solanum stenotomum*	delta(12)-fatty-acid desaturase	GenBank	XP_049402823.1	383	Complete	Yes
SsFAD2-2	*Solanum stenotomum*	delta(12)-fatty-acid desaturase	GenBank	XP_049402822.1	383	Complete	Yes
StoFAD2	*Senna tora*	delta(12)-fatty-acid desaturase	GenBank	KAF7818956.1	383	Complete	Yes
StuFAD2-1	*Solanum tuberosum*	delta(12)-fatty-acid desaturase	GenBank	XP_006349589.1	384	Complete	Yes
StuFAD2-2	*Solanum tuberosum*	delta(12)-fatty-acid desaturase	GenBank	XP_006365798.1	383	Complete	Yes
StuFAD2-3	*Solanum verrucosum*	delta(12)-fatty-acid desaturase	GenBank	XP_049363758.1	383	Complete	Yes
StuFAD2-4	*Solanum verrucosum*	delta(12)-fatty-acid desaturase	GenBank	XP_049363760.1	383	Complete	Yes
StuFAD2-5	*Solanum verrucosum*	delta(12)-fatty-acid desaturase	GenBank	XP_049365225.1	384	Complete	Yes
TcFAD2-1	*Theobroma cacao*	delta(12)-fatty-acid desaturase	GenBank	XP_007014826.2	377	Complete	Yes
TcFAD2-2	*Theobroma cacao*	delta(12)-fatty-acid desaturase	GenBank	EOY32445.1	377	Complete	Yes
ThFAD2-1	*Tarenaya hassleriana*	delta(12)-fatty-acid desaturase	GenBank	XP_010557947.1	383	Complete	Yes
ThFAD2-2	*Tarenaya hassleriana*	delta(12)-fatty-acid desaturase	GenBank	XP_010520034.1	437	Complete	Yes
ThFAD2-3	*Tarenaya hassleriana*	delta(12)-fatty-acid desaturase	GenBank	XP_010520035.1	383	Complete	Yes
TseFAD2	*Triadica sebifera*	delta(12)-fatty-acid desaturase	GenBank	ABI96919.1	381	Complete	Yes
TstFAD2	*Trifolium subterraneum*	uncharacterized protein	GenBank	GAU23423.1	384	Complete	Yes
TsuFAD2	*Turnera subulata*	delta(12)-fatty-acid desaturase	GenBank	KAJ4838139.1	385	Complete	Yes

TwFAD2-1	*Tripterygium wilfordii*	delta(12)-fatty-acid desaturase	GenBank	XP_038702086.1	381	Complete	Yes
TwFAD2-2	*Tripterygium wilfordii*	delta(12)-fatty-acid desaturase	GenBank	XP_038697417.1	383	Complete	Yes
VrFAD2-1	*Vitis riparia*	delta(12)-fatty-acid desaturase	GenBank	XP_034687341.1	376	Complete	Yes
VrFAD2-2	*Vitis vinifera*	delta(12)-fatty-acid desaturase	GenBank	XP_002285640.1	376	Complete	Yes
VrFAD2-3	*Vitis vinifera*	delta(12)-fatty-acid desaturase	GenBank	RVW48567.1	376	Complete	Yes
XsFAD2-1	*Xanthoceras sorbifolium*	uncharacterized protein	GenBank	KAH7554281.1	383	Complete	Yes
XsFAD2-2	*Xanthoceras sorbifolium*	delta(12)-fatty-acid desaturase	GenBank	AGO32050.1	383	Complete	Yes
Insect							
AdD12Des	*Acheta domesticus*	delta(12)-fatty-acid desaturase	GenBank	ABY26957.1	357	Complete	Yes
AprFAD2-1	*Aleyrodes proletella*	——[e)]	Transcriptome	——[e)]	381	Complete	Yes
AprFAD2-2	*Aleyrodes proletella*	——[e)]	Transcriptome	——[e)]	378	Complete	Yes
ApsFAD2	*Aleuroclava psidii*	——[e)]	Genome	——[e)]	380	Complete	Yes
AsFAD2-1	*Aleurocanthus spiniferus*	——[e)]	Transcriptome	——[e)]	381	Complete	Yes
AsFAD2-2	*Aleurocanthus spiniferus*	——[e)]	Transcriptome	——[e)]	382	Complete	Yes
BtFAD2-1	*Bemisia tabaci*	delta(12)-fatty-acid desaturase	GenBank	OQ291260	382	Complete	Yes
BtFAD2-10	*Bemisia tabaci*	delta(12)-fatty-acid desaturase	GenBank	OQ291269	402	Complete	Yes
BtFAD2-11	*Bemisia tabaci*	delta(12)-fatty-acid desaturase	GenBank	OQ291270	404	Complete	Yes
BtFAD2-12	*Bemisia tabaci*	delta(12)-fatty-acid desaturase	GenBank	OQ291271	371	Complete	Yes
BtFAD2-13	*Bemisia tabaci*	delta(12)-fatty-acid desaturase	GenBank	OQ291272	593	Complete	Yes
BtFAD2-2	*Bemisia tabaci*	delta(12)-fatty-acid desaturase	GenBank	OQ291261	436	Complete	Yes
BtFAD2-3	*Bemisia tabaci*	delta(12)-fatty-acid desaturase	GenBank	OQ291262	387	Complete	Yes
BtFAD2-4	*Bemisia tabaci*	delta(12)-fatty-acid desaturase	GenBank	OQ291263	390	Complete	Yes

BtFAD2-5	*Bemisia tabaci*	delta(12)-fatty-acid desaturase	GenBank	OQ291264	373	Complete	Yes
BtFAD2-6	*Bemisia tabaci*	delta(12)-fatty-acid desaturase	GenBank	OQ291265	379	Complete	Yes
BtFAD2-7	*Bemisia tabaci*	delta(12)-fatty-acid desaturase	GenBank	OQ291266	375	Complete	Yes
BtFAD2-8	*Bemisia tabaci*	delta(12)-fatty-acid desaturase	GenBank	OQ291267	599	Complete	Yes
BtFAD2-9	*Bemisia tabaci*	delta(12)-fatty-acid desaturase	GenBank	OQ291268	381	Complete	Yes
ClD12Des	*Chauliognathus lugubris*	delta(12)-fatty-acid desaturase	GenBank	AFJ66832.1	348	Complete	Yes
DcFAD2-1	*Dialeurodes citri*	——[e)]	Transcriptome	——[e)]	353	Partial, lacks N-terminus	Yes
DcFAD2-2	*Dialeurodes citri*	——[e)]	Transcriptome	——[e)]	305	Partial, lacks N-terminus	Yes
NvD12Des1	*Nasonia vitripennis*	stearoyl-CoA desaturase 5	GenBank	XP_001599836.1	362	Complete	Yes
NvD12Des2	*Nasonia vitripennis*	stearoyl-CoA desaturase 5	GenBank	XP_001599873.1	362	Complete	Yes
TcD12Des	*Tribolium castaneum*	delta(12)-fatty-acid desaturase	GenBank	NP_001137206.1	358	Complete	Yes
TvFAD2	*Trialeurodes vaporariorum*	——[e)]	Genome	——[e)]	387	Complete	Yes

[a)]The names of these genes have been adjusted to a uniform nomenclature.

[b)]Annotation represents the protein information in the GenBank database (http://www.ncbi.nlm.nih.gov/). Some annotation information has been adjusted for clearer presentation.

[c)]Gene ID represents the gene accession number of the GenBank database (http://www.ncbi.nlm.nih.gov/).

[d)]Whether or not these genes were used in the phylogenetic analysis in Figure 1C.

[e)]Aleyrodinae FAD2 sequences identified by tBLASTn without cloning validation. Detailed sequence information is provided in Table S3, Supporting Information.

Table S3. Detailed information regarding Aleyrodinae FAD2 sequences (excel file)
